# Multiscale Simulation Guided Electric Field-Enhanced Ammonia Catalytic Cracking

**DOI:** 10.1021/acscatal.5c01829

**Published:** 2025-04-24

**Authors:** Pragyansh Singh, Qiang Li, Yilang Liu, Fanglin Che

**Affiliations:** Department of Chemical Engineering, University of Massachusetts, Lowell, Massachusetts 01854, United States

**Keywords:** hydrogen, ammonia, density functional theory, microkinetic model, electric fields

## Abstract

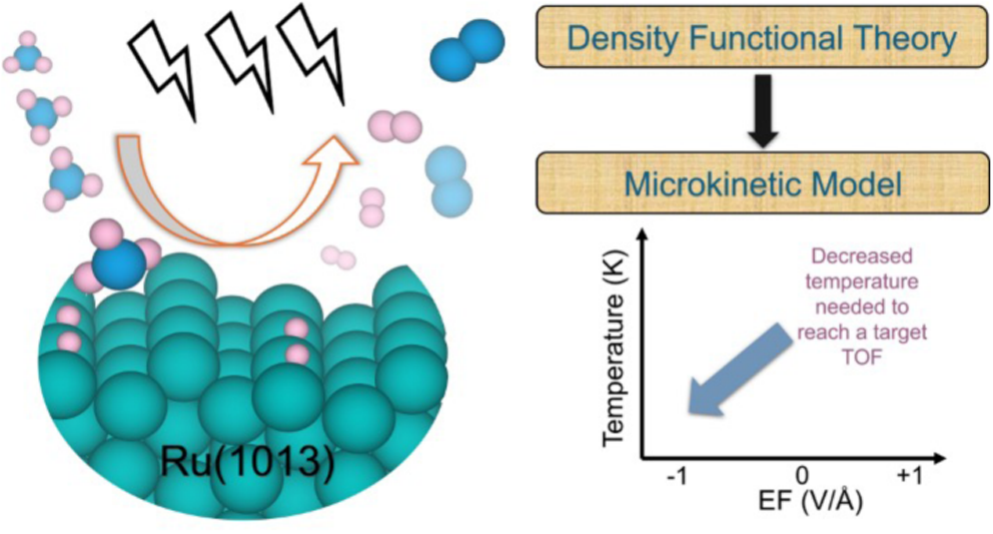

Ammonia catalytic cracking offers an efficient solution for hydrogen production, storage, and distribution, making it ideal for onboard hydrogen generation in maritime propulsion systems when integrated with fuel cells. However, conventional heating methods, even with highly active ruthenium (Ru) catalysts, require high temperatures to achieve satisfactory performance, posing a challenge for industrial implementation. A promising strategy to address this limitation is the application of strong external electric fields, which can lower the temperature requirement through interactions between fields and the dipoles of polarized species during ammonia cracking. To explore such a field-dipole effect, we developed a multiscale simulation framework that integrates density functional theory (DFT) calculations with microkinetic modeling. This framework provides mechanistic insights, identifies key rate-limiting steps, and optimizes conditions for field-enhanced ammonia catalytic cracking over Ru. Our results show that at 673 K, applying a −1 V/Å negative electric field dramatically increases the turnover frequency from 0.03 s^–1^ (zero field) to 1435.2 s^–1^. Similarly, at a higher temperature of 823 K, the negative electric field enhances the turnover frequency by 4 orders of magnitude compared to the no field conditions. In addition, applying a −1 V/Å electric field reduces the operating temperature from 750 K (zero field) to 586 K while maintaining a given turnover frequency (e.g., 5 s^–1^). Sensitivity analysis further identifies NH dehydrogenation over Ru(1013) as the rate-limiting step across various electric fields and temperatures. This multiscale model enhances the understanding of field-enhanced catalysis, offering valuable insights into the development of more efficient hydrogen production processes.

## 1 Introduction

Hydrogen (H_2_) presents a carbon-free alternative to fossil fuels.^1−4^ However, challenges in storage and safe transportation continue to limit the widespread adoption of a hydrogen economy.^5−9^ Recent advancements emphasize the potential of chemical hydrogen storage, where H_2_ is stored within a chemical compound and released through catalytic cracking, offering a promising solution to storage and transportation challenges.^10−13^ Several hydrogen-containing compounds have been proposed for storage and release, including ammonia,^14,15^ methane,^16^ ammonia-borane,^17,18^ methanol,^11,19^ and water.^20−23^ Among these options, ammonia^24^ (NH_3_) emerges as a good carbon-free candidate possessing high H_2_ density, ease of liquefication, and leveraging existing infrastructure for transportation.^25,26^ However, even with the highly active ruthenium (Ru) catalysts, NH_3_ cracking for H_2_ generation requires high temperatures (e.g., >1000 K) to achieve complete conversion. This is due to the substantial kinetic barriers associated with the initial N–H bond cleavage and subsequent N≡N bond formation.^27,28^

To reduce the reaction temperature and overcome the kinetic limitations of NH_3_ cracking in an electrified modular setup, the application of external electric fields presents a promising solution. An external electric field can reorganize the electronic orbitals of polarized species, modify reaction mechanisms, lower kinetic barriers for potential rate-limiting steps, and enhance energy efficiency in catalytic NH_3_ cracking.^29−37^ Several experimental studies have investigated the impact of electric fields on ammonia catalytic cracking/synthesis. Maslova et al.^38^ explored electric field-assisted NH_3_ cracking over various Ce-based catalysts in a packed bed reactor, focusing on key parameters such as applied current, NH_3_ concentration, and residence time. Their findings revealed that a 1 wt % Fe/CeO_2_ catalyst exhibited the highest activity, achieving a 22% conversion at 4 mA, with 0.18 mmol of NH_3_ converted per kJ of energy consumed at 823 K. Triviño et al.^39^ reported that electric field-enhanced ammonia synthesis (the dipole moment changes  of ammonia synthesis is 0.3 eÅ) increases energy efficiency to 36.3 gNH_3_/kWh and enhances reaction rates by up to a 10-fold compared to conventional heating. Thus, ammonia cracking and synthesis reactions are sensitive to the field-dipole interaction effect, and their energy efficiencies can be significantly enhanced under electric fields.

Understanding electric field effects in heterogeneous catalysis under operando conditions and designing field-enhanced catalytic reactors based on fundamental principles, rather than trial-and-error, to achieve maximum energy efficiency remain challenging. The lack of well-defined solid–gas interfaces and limited high-resolution operando spectroscopy hinder the characterization of field-dipole interactions and their impact on catalytic mechanisms, thermodynamics, and kinetics.^40,41^

The above challenges emphasize the necessity of theoretical approaches, such as density functional theory (DFT), to understand how electric fields modify electronic structures, binding energies, and reaction pathways at the atomic level.^42−52^ Chen et al.^53^ used the linear combination of atomic dipole (LCAD) method with ab initio calculations to accurately predict dipole moments for molecules of varying sizes, achieving small errors relative to experiments while effectively capturing the influence of electric fields. Deshlahra et al.^54^ combined DFT and experiments to study electric field effects on CO chemisorption on Pt(111), revealing that electronic differences between atop and fcc sites lead to distinct electric field responses, with a site shift from atop to fcc below −0.19 V/Å and a Stark tuning rate of 44.4 cm^–1^ V^–1^ Å. Vaissier et al.^55^ enhanced the catalytic efficiency of a Kemp eliminase enzyme by 43-fold through molecular dynamics-guided mutations that optimize electric fields and substrate positioning, demonstrating the potential of field-driven enzyme design. Li et al.^46^ showed that conserved residues in enzymes generate nonlocal electric fields that preorganize transition states, reducing reorganization energy in line with Warshel’s paradigm. Their later work^45^ on Ga_4_L_6_^12-^ nanocages found that replacing Ga with In reduced solvent reorganization energy and improved field alignment with the transition state. Welborn et al.^47^ further showed that encapsulated water in the same capsule can stabilize transition states via internal electric fields. Recently, Li et al.^48^ developed TinkerModeller, a tool that simplifies the use of polarizable force fields and introduces an efficient electric field estimation module, making electrostatic modeling more accessible. However, the above atomic- or molecular-scale calculations alone cannot fully capture the impact of electric fields on the catalytic performance or align with experimental conditions. To bridge this gap, our work integrates DFT with microkinetic modeling (MKM) to identify rate-limiting steps and quantify the field-dependent catalytic activity.

In this study, we integrated the DFT and MKM to investigate the effects of electric field on NH_3_ cracking over Ru(0001) (the most thermodynamic favorable flat surface) and Ru(1013) (the most favorable kink surface with the most active B5 site^56^). Initially, DFT calculations were conducted to obtain the intrinsic kinetics and thermodynamics of the decomposition network. Subsequently, a DFT-based microkinetic model coupled with sensitivity analysis was employed to assess reaction kinetics and pinpoint potential rate-limiting steps for NH_3_ cracking under various electric field conditions. Our findings reveal that a negative electric field can enhance the overall kinetic activity (as measured by turnover frequency, TOF). Moreover, a systematic comparison of field-driven changes in energetics of intermediates and transition states suggests the critical importance of electric field in controlling the thermodynamic and kinetic properties of polarized surface species, thereby optimizing catalytic performance. These theoretical findings highlight the profound impact of electric field in shifting the reaction mechanism and pave the way for optimizing the reaction parameters to maximize the reaction rates of NH_3_ cracking into H_2_.

## 2 Methods

### 2.1 Computational Details

Density functional theory (DFT) calculations were carried out using the Vienna ab initio simulation package (VASP).^57,58^ The generalized gradient approximation (GGA) with the Perdew–Burke–Ernzerhof (PBE) exchange–correlation functional was employed.^59−61^ Valence states were expanded in plane waves with a cutoff energy of 500 eV, while core electrons were treated using the projector-augmented wave (PAW) method to solve the Kohn–Sham equation.^62^ The optimized lattice constant of ruthenium was 2.726 Å (*c*/*a* = 1.5), closely matching experimental and previous theoretical values.^63^ A Monkhorst–Pack *k*-point mesh of (3 × 3 × 1) was used for the Ru(0001) and Ru(1013) surfaces (Figure S1).^64^ During geometry optimization, the bottom two layers were fixed at their bulk positions, while the top two layers were allowed to relax. Convergence criteria were set to 10^–5^ eV for energy and 0.03 eV/Å for forces. Electric field effects were simulated using the Neugebauer and Scheffler approach,^65^ which generates a uniform electric field without altering the total electrons in the supercell. For “field emission” effects,^66^ a vacuum depth of ∼11 Å was applied, ensuring that the charge density in the vacuum remained below 0.001 e/Å^3^, minimizing Gibbs oscillations from the plane-wave cutoff. Complementary spin-polarized calculations investigated the field effects on surface magnetization. Both Ru(0001) and Ru(1013) exhibited no detectable spin polarization under negative, neutral, and positive electric fields (−1 to +1 V/Å), confirming the preserved nonmagnetic character of ruthenium across applied fields.

To determine the most stable configurations of adsorbed species, various adsorption sites were investigated on both Ru surfaces (Figures S2–S15). Subsequently, the influence of the electric field on the adsorption configurations and energies was evaluated (Figures S16 and S17). Coadsorption energetics and configurations of reaction intermediates were modeled based on the most stable configurations of each adsorbate (Figures S18–S29), and field-dependent reaction energies for elementary steps were calculated (Figures S30 and S31). Transition states were initially identified using the climbing image-nudged elastic band (CI-NEB) method^67^ to obtain approximate structures, followed by full optimization using the dimer method (Figures S32–34) (Table S1).^68^ We also calculated the vibrational frequency of the transition state to confirm the presence of the only one imaginary frequency along the corresponding reaction coordinate. Moreover, electronic charge distribution analysis was performed by using the Bader charge method (Figure S35). To understand the sensitivity of the field effects, calculations for a representative elementary step were extended to other potential ammonia cracking catalytic surfaces (Figure S36). More comprehensive details regarding the computational methodology and DFT modeling parameters are provided in the Supporting Information (Section S1).

### 2.2 Microkinetic Modeling

A microkinetic model (MKM) was developed to simulate NH_3_ cracking with and without electric fields in a plug flow reactor (PFR) (Table S2). The reactor was modeled as an isothermal steady-state system with no axial dispersion and negligible pressure drops. The governing differential equations accounted for both forward and reverse reaction rates, describing the consumption of gas-phase and surface species. Further details on the elementary reaction steps and input parameters (Table S3) for the MKM are provided in Section S2. All kinetic parameters were derived using the Python Multiscale Thermochemistry Toolbox (pMuTT)^69^ under the harmonic oscillator approximation, and the MKM simulations were performed using the Cantera package.^70^

## 3 Results and Discussion

### 3.1 NH_3_ Cracking Reaction Networks

**Figure 1:**
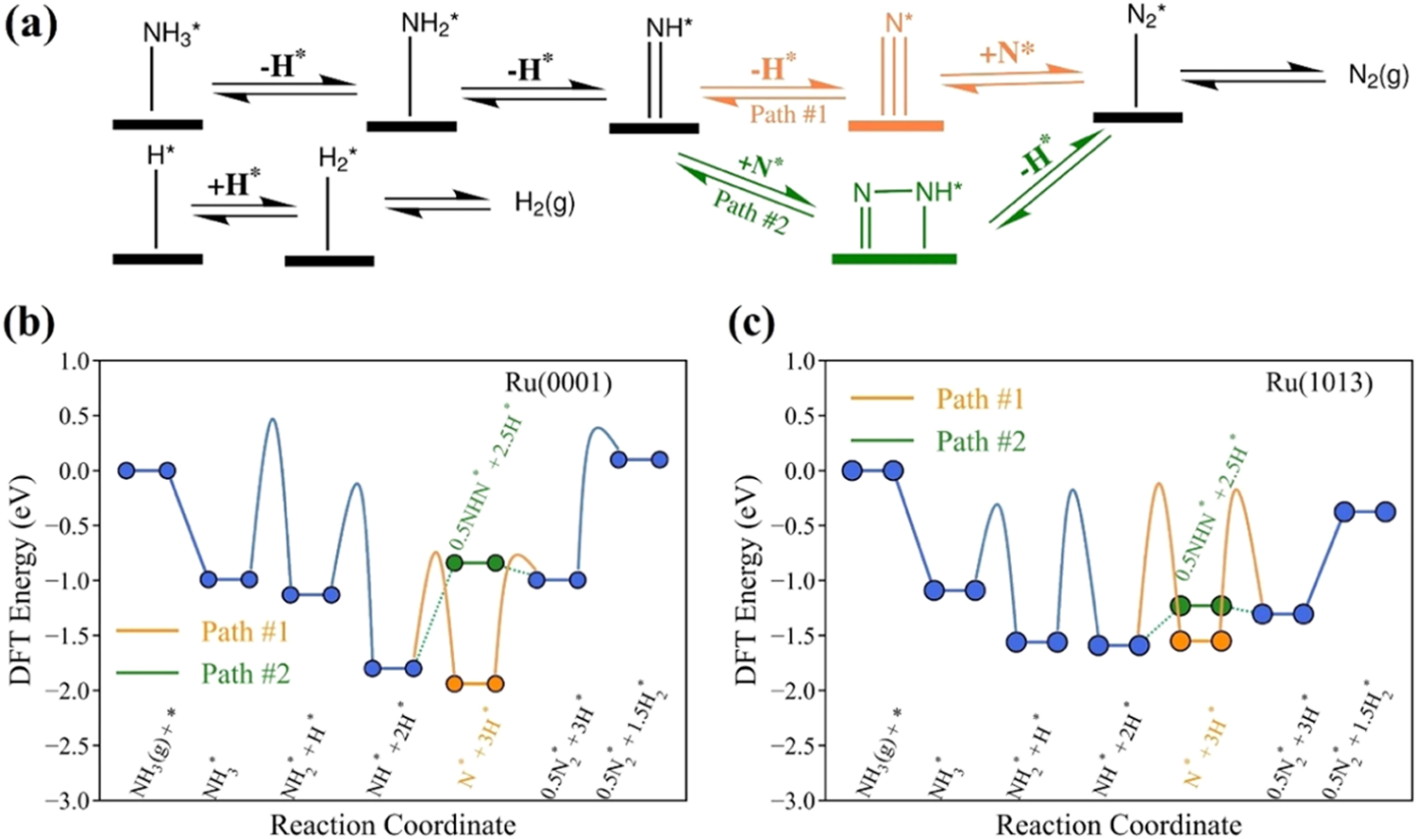
(a) Two predominant pathways of NH_3_ cracking. The corresponding potential energy diagram of NH_3_ cracking over (b) Ru(0001) and (c) Ru(1013) surfaces.

As illustrated in Figure 1a, we compared two predominant pathways for NH_3_ decomposition, with the N≡N bond formation from 2N* (Path 1) and NH*–N* (Path 2) couplings, respectively. A thermodynamic comparative analysis of these two pathways on the Ru(0001) and Ru(1013) surfaces is shown in Figure 1b,c. Ru(0001) is the most thermodynamically favorable flat surface for Ru, while Ru(1013) has the most active B5 site for ammonia cracking. The reaction energy profiles indicate that NH dehydrogenation (Path 1) is energetically more favorable than N–NH formation (Path 2) by 1.1 eV on Ru(0001) and 0.32 eV on Ru(1013). In addition, overall N_2_* and H_2_* formation over Ru(1013) is favored compared to Ru(0001) by 0.48 eV, implying that NH_3_ cracking thereby is thermodynamically preferred over Ru(1013). The transition-state barrier study over the most favorable pathway (Path 1) suggests that N_2_ formation (2N* → N_2_* + *) is the potential rate-limiting step on the Ru(0001) surface with a kinetic barrier of 2.18 eV, whereas over the Ru(1013) surface, NH dehydrogenation (NH* + * → N* + H*) emerges as the potential rate-limiting step with a kinetic barrier of 1.47 eV, suggesting that NH_3_ cracking is also kinetically preferred over Ru(1013). These rate-limiting steps will be further validated using MKM in the next sections.

### 3.2 Effect of Temperature on NH_3_ Cracking Kinetics

**Figure 2:**
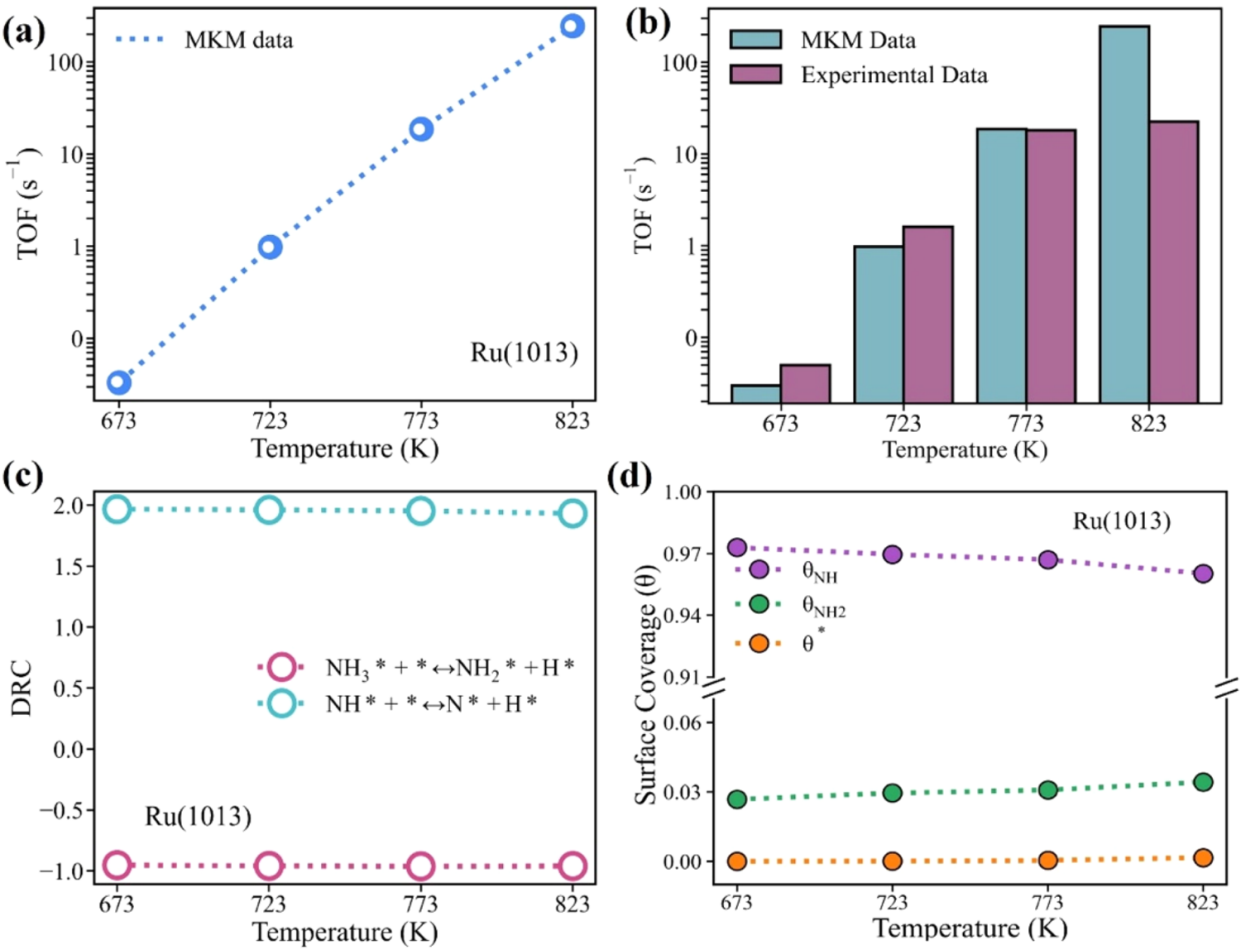
(a) MKM predicted TOF of NH_3_ cracking over Ru(1013) as a function of temperature. (b) Benchmarking the TOF from our model with respect to the reported experimental performance of the Ru catalyst at 673,^72^ 723,^73^ 773,^74^ and 823 K.^75^ (c) Degree of rate control analysis for NH_3_ cracking over Ru(1013). (d) Temperature dependence of steady-state surface coverages for key species during NH_3_ cracking on the Ru(1013) surface.

The steady-state turnover frequencies (TOFs) for thermal NH_3_ cracking on the active Ru(1013) surface were calculated with the DFT-derived energetics of the most favorable reaction pathway (Path 1) in microkinetic modeling (MKM, Figure 2a). The temperature range of 673–823 K, extensively documented in the experimental literature for ammonia decomposition,^71^ was selected as the domain for this investigation. Our results show that the predicted TOF monotonically increases from 0.03 to 245 s^–1^ as the temperature increases from 673 to 823 K. To validate the MKM-derived TOF estimations on the Ru surface, experimental data reported by Lucentini et al.^71^ were adopted as a benchmark. The paper reported experimental TOF values across diverse Ru catalysts, providing a reliable reference data set for benchmarking computational models. Figure 2b presents a comparative analysis between the model’s predictions and the experimental data at specific temperature points: 673, 723, and 773 K. The model predicted TOFs of 0.03, 0.98, and 18.64 s^–1^ at these respective temperatures, which align well with the corresponding experimental values of 0.05, 1.6, and 18.1 s^–1^, respectively.

To identify the rate-limiting step of NH_3_ cracking on Ru(1013) across different temperatures, a degree of rate control (DRC) analysis^76,77^ was conducted (Figure 2c). This analysis quantifies the contribution of each elementary step to the overall reaction rate by perturbing its rate constant by 1% while keeping its equilibrium constant and the rate constants of other elementary steps fixed. The elementary step with the highest positive DRC value is identified as the potential rate-limiting step, while the one with a negative DRC value inhibits the reaction. Our DRC results show that the NH dehydrogenation step consistently exhibited a positive DRC value of ∼2.0 across all examined temperatures (673–823 K), confirming its rate-limiting nature. This is further validated by the surface coverage analysis that the surface dominant intermediate is NH* with almost full surface coverage (Figure 2d) and its dehydrogenation has the relatively higher activation energy compared to other elementary steps (Figure 1c). Since in catalytic reactions rate-limiting steps can be characterized by the accumulation of species on the surface due to their faster production than their consumption rates. Therefore, the presence of high surface coverage of NH* observed for our case verifies NH dehydrogenation as the rate-limiting step.^78^

In contrast, NH_3_ dehydrogenation maintained a negative DRC value of ∼−1.0, indicating that this step suppresses the NH_3_ cracking reaction over Ru(1013). To understand this, we performed a sensitivity analysis, coupled with the analysis of surface coverage and reaction rates.^79^ Via an increase in the forward rate constant of NH_3_ dehydrogenation by 1% at 673 K, it decreases the fraction of free active sites (θ*) by approximately 1% (Figure S37) and it further decreased the rate of rate-limiting step (RLS, NH* + * → N* + H*, eqs 1 and 2) by around 1% (Figure S38). These findings collectively suggest that the inhibition effect of the NH_3_ dehydrogenation step is correlated with the reduction in the availability of free active sites, which in turn slows down the rate of RLS, thereby decreasing the overall reaction rate. This trend is consistently observed over the entire examined temperature range (673–823 K)
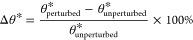
1
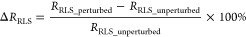
2

### 3.3 Reaction Networks with Electric Fields

**Figure 3:**
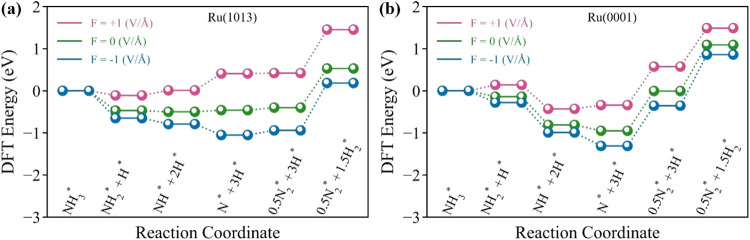
Energy diagram of the NH_3_ cracking on the (a) Ru(1013) and (b) Ru(0001) surfaces under different electric fields.

**Figure 4:**
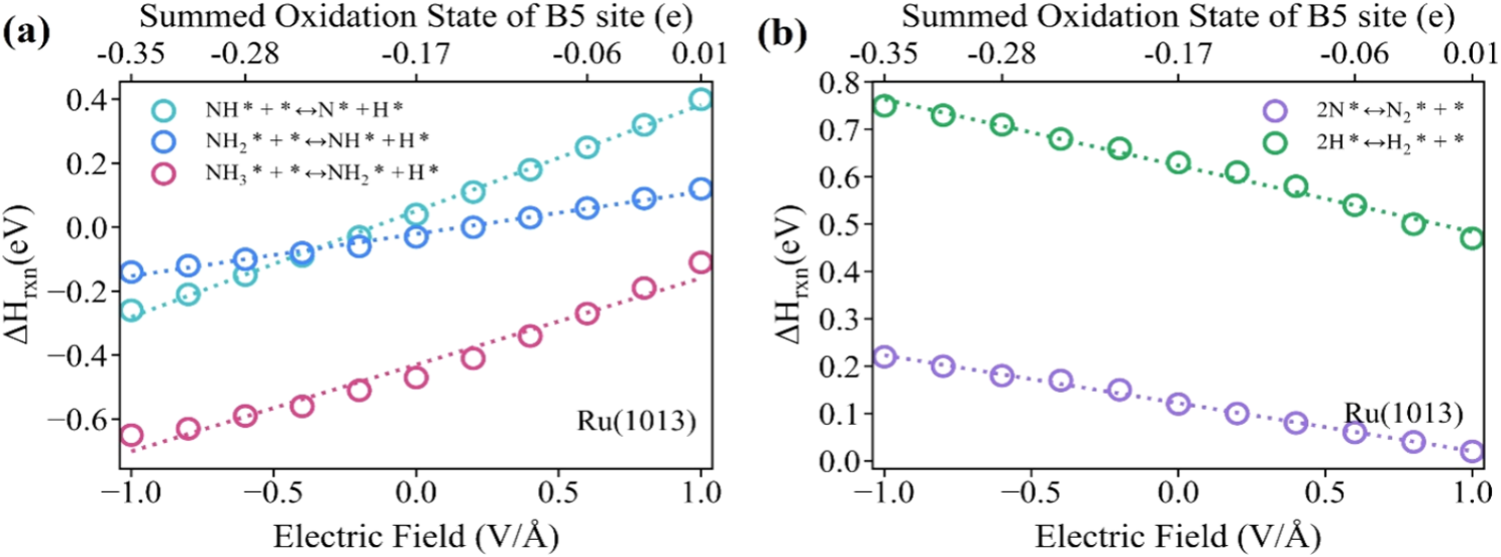
Reaction energies of (a) bond dissociation and (b) bond formation steps on Ru(1013) as a function of applied electric field and the oxidation state of the B5 sites.

We then examined the effect of electric fields on the thermodynamic properties of NH_3_ cracking over the most favorable Ru(1013) surface. Figure 3a illustrates the energy diagram of the most favorable reaction pathway (path 1) under different electric fields. Our results indicate that, compared to the zero-field case, applying a −1 V/Å electric field lowers the overall reaction energy of NH_3_ decomposition (NH_3_* → 0.5N_2_* + 1.5H_2_*) over Ru(1013) by 0.35 eV. In contrast, a positive electric field has the opposite effect, making the reaction more endothermic by 0.58 eV at 1 V/Å. A similar trend is observed on the less reactive Ru(0001) surface with reaction energies reduced by 0.23 eV at −1 V/Å and increased by 0.4 eV at +1 V/Å (Figure 3b). Interestingly, the negative electric fields enhance bond dissociation steps, particularly dehydrogenation steps of NH_x_* (Figure 4a), while positive electric fields favor bond formation, such as N–N and H–H bond formation (Figure 4b).

**Figure 5:**
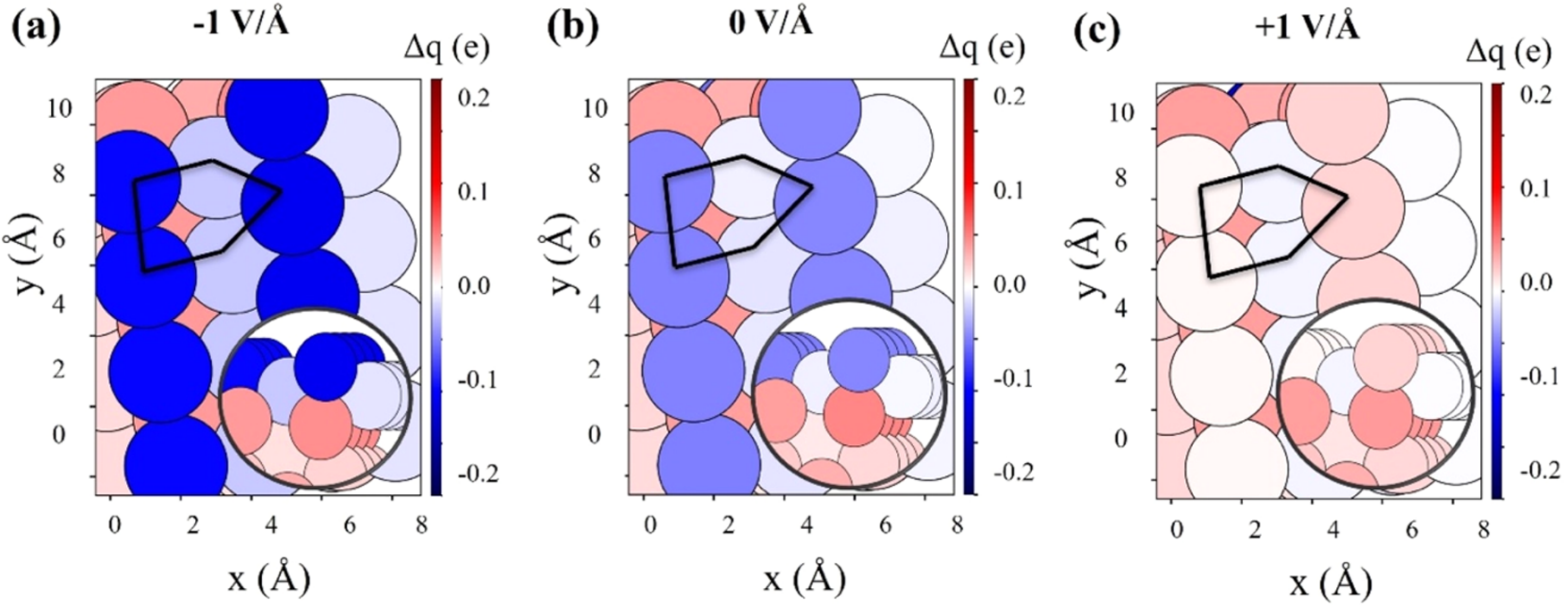
Top and side views (inset) of the Bader charge distribution on the Ru(1013) surface, highlighting B5 sites (marked with pentagons) under the applied electric field conditions of (a) −1 V/Å, (b) 0 V/Å, and (c) +1 V/Å. The mentioned Δ*q* represents the net charge on the atom. A positive value indicates a positively charged atom, and vice versa.

To understand the properties of the bond-forming and bond-dissociating elementary steps during NH_3_ cracking under various external electric fields, we examined the electronic structure of the Ru(1013) surface. The electronic charge distribution at the active B5 site^80^ was quantitatively assessed using the Bader charge analysis under various electric field strengths (Figure 5a–c). The cumulative oxidation state of the B5 site exhibited a monotonic increase from −0.35 to 0.01 e as the applied field increases from −1 to +1 V/Å. Negative electric fields create an electron-rich environment at the B5 site, making it easier to break N–H bonds by supplying extra electrons to compensate for those lost during bond cleavage. In contrast, positive electric fields reduce the electron density at the surface, making it more electrophilic and drawing electrons away from adsorbed molecules. To counteract this electron loss, molecules are more likely to form new bonds, favoring reactions like N_2_ and H_2_ formations. Comparative Bader charge analysis on the flat Ru(0001) surface (Figure S35) highlighted a key difference: unlike the uniform charge distribution on Ru(0001), the Ru(1013) surface exhibits pronounced charge localization at its kinked B5 sites. These sites accumulate more negative charge under a −1 V/Å field, correlating with the enhanced ammonia decomposition activity observed on Ru(1013) relative to Ru(0001) (Figures S30 and S31). Interestingly, we found that the electric field sensitivity of catalytic reactions is primarily determined by surface geometry rather than the metal element, as demonstrated by the greater dipole moment and polarizability on the kinked Ru(1013) surface compared to flat Ru(0001) and similar field responses across different elements with the same (1013) geometry such as Co, Ru, and Re (Figure S36).

**Figure 6:**
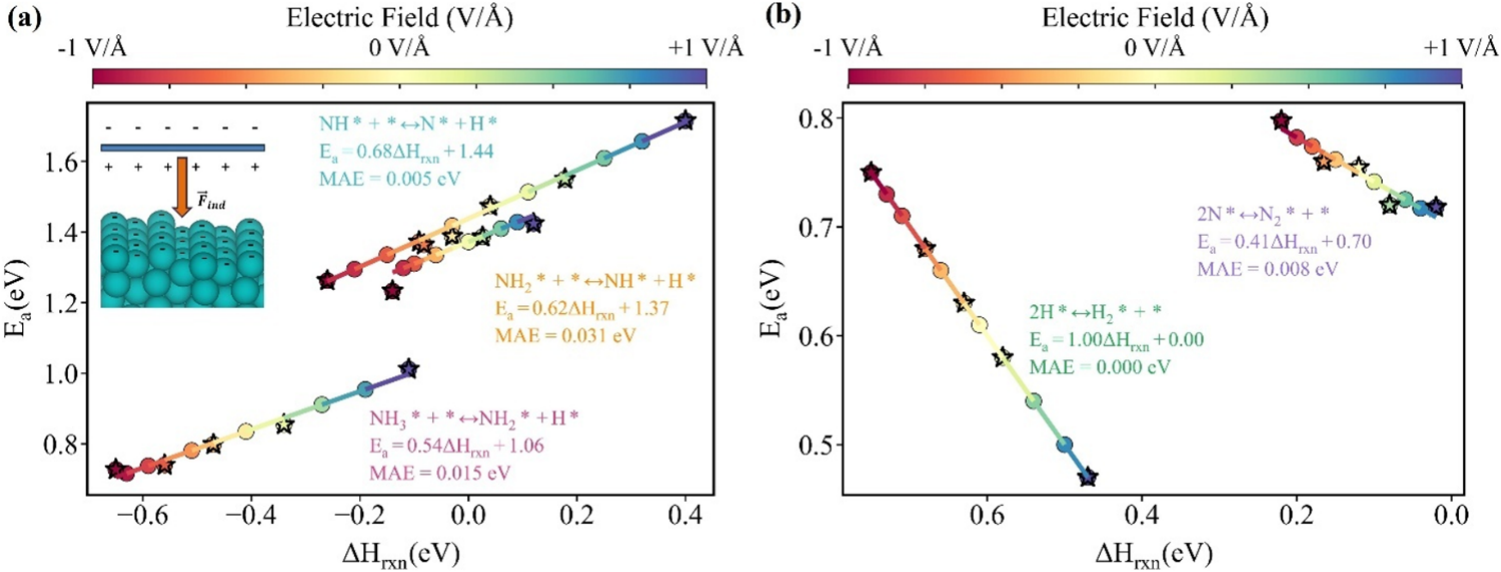
Reaction energies and activation barriers of (a) bond-dissociating and (b) bond-forming steps in NH_3_ cracking under various electric fields. The inset in panel (a) shows how we theoretically applied a negative field. DFT calculations were performed to determine activation barriers at −1, −0.4, 0, +0.4, and +1 V/Å electric fields, while values for other field strengths were extrapolated using the established BEP correlations. The stars in both plots represent the DFT-calculated data points.

We further examined the impact of electric fields on the kinetic barriers of NH_3_ cracking on Ru(1013) (Table S1 and Figure S32). Since the reaction energetics and activation barriers under different electric fields follow the Brønsted–Evans–Polanyi (BEP) relationship,^49,52^ as activation barriers of elementary steps exhibit a linear correlation with reaction energies under varying field strengths (Figure 6a,b). We computed activation barriers at +1, +0.4, 0, −0.4, and −1 V/Å electric fields to validate the BEP relationship, while the remaining barriers were estimated by using the established BEP correlation. Negative electric fields promote bond dissociation, significantly lowering activation barriers for NH*_x_* dehydrogenation with decreasing field strength from +1 to −1 V/Å: NH_3_ (1.01 → 0.73 eV), NH_2_ (1.42 → 1.23 eV), and NH (1.71 → 1.26 eV). Conversely, positive electric fields facilitate bond formation, as seen in N_2_ formation, where the activation barrier decreases from 0.79 to 0.71 eV as the electric field strength increases from −1 to +1 V/Å. No saddle point was observed for the H_2_ formation step during transition-state calculation (Figure S33). The endothermic reaction energy of H_2_ formation exhibits a similar trend to that of N_2_ formation; it becomes more thermodynamically favorable under a positive electric field; with the field increasing from −1 to +1 V/Å, the energy difference decreases from 0.75 to 0.47 eV. Since the rate-limiting step is NH dissociation (Figure 2c), the negative electric field can potentially promote the ammonia decomposition. Our previous work^81^ used graph neural network (GNN)–accelerated DFT calculations to study the effects of electric fields on Ru- and Fe-catalyzed ammonia syntheses. In contrast to ammonia decomposition, a positive electric field enhances NH_3_ formation by decreasing activation barriers by 0.11 and 0.25 eV at +1 V/Å compared to no electric fields and negative fields of −1 V/Å and promotes associative pathways (e.g., N_2_H* formation) over the direct N≡N bond dissociation on Ru(1013) at positive fields above 0.6 V/Å.

The activation energy under electric fields can also be described by a second-order Taylor expansion involving changes in dipole moment and polarizability (eq S5), and comparison of dipole changes between activation barriers and reaction energetics (Figure S34) shows that electric fields affect reaction energies more strongly than activation barriers—consistent with BEP correlations exhibiting a slope less than one.

### 3.4 Effects of Electric Fields and Temperature on NH_3_ Cracking Kinetics

Our DFT results reveal that negative electric fields facilitate bond dissociations, whereas positive electric fields favor bond formations. Since both bond dissociation (N···H) and formation steps (N···N, H···H) are involved in the NH_3_ cracking network, we extended our MKM model by incorporating DFT-derived energetics under various electric field strengths to quantitatively assess the impact of electric fields on reaction mechanisms (rate-limiting steps) and overall catalytic activity as described by turnover frequency (TOF).

**Figure 7:**
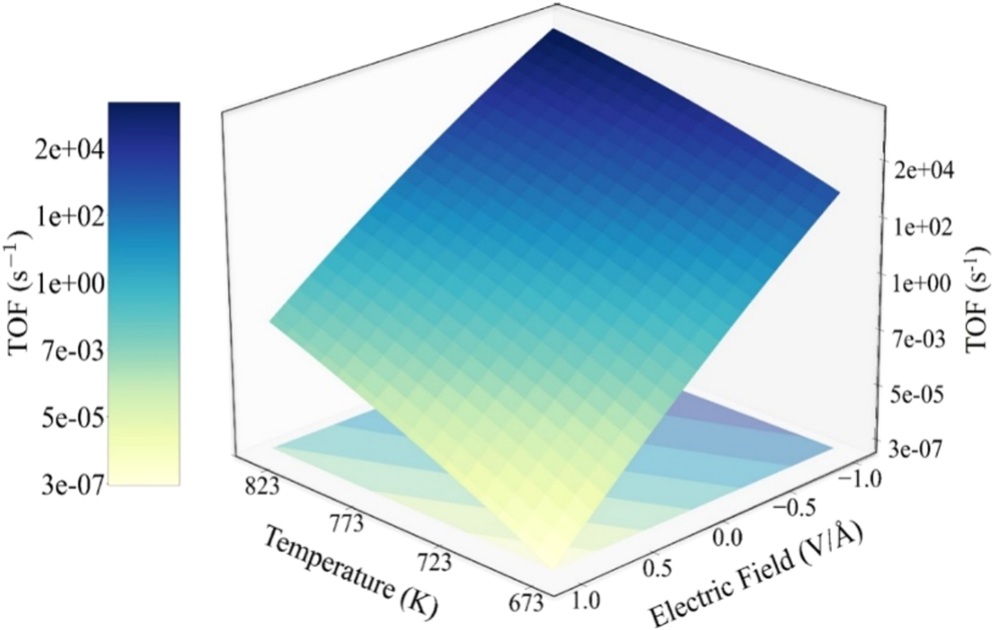
Effects of temperature and electric field on the TOF of NH_3_ cracking on Ru(1013).

As shown in Figure 7, the MKM results indicate that the TOF increases with increasing temperature and applying negative electric fields, while positive electric fields suppress the activity. At a constant temperature of 673 K (the thermodynamic equilibrium temperature for complete ammonia cracking conversion),^82^ the TOF varies significantly with electric field strength, increasing from ∼1.34 × 10^–6^ s^–1^ at +1 V/Å to ∼3.3 × 10^–2^ s^–1^ in a zero-field case (0 V/Å) and further reaching ∼1.43 × 10^3^ s^–1^ at −1 V/Å, demonstrating that a negative electric field (−1 V/Å) enhances TOF by approximately 4 orders of magnitude compared to the zero-field case. Furthermore, to achieve a target turnover frequency, e.g., approximately 5 s^–1^, the application of a −1 V/Å electric field allows a temperature reduction from 750 (zero field) to 586 K. These observations highlight the potential of negative electric fields to enhance catalytic activity and reduce the required operating temperature of NH_3_ cracking. This enhancement is attributed to negative-field-induced electron enrichment at the B5 active sites, which facilitates the rate-limiting step of NH bond dissociation (Figure 5).

**Figure 8:**
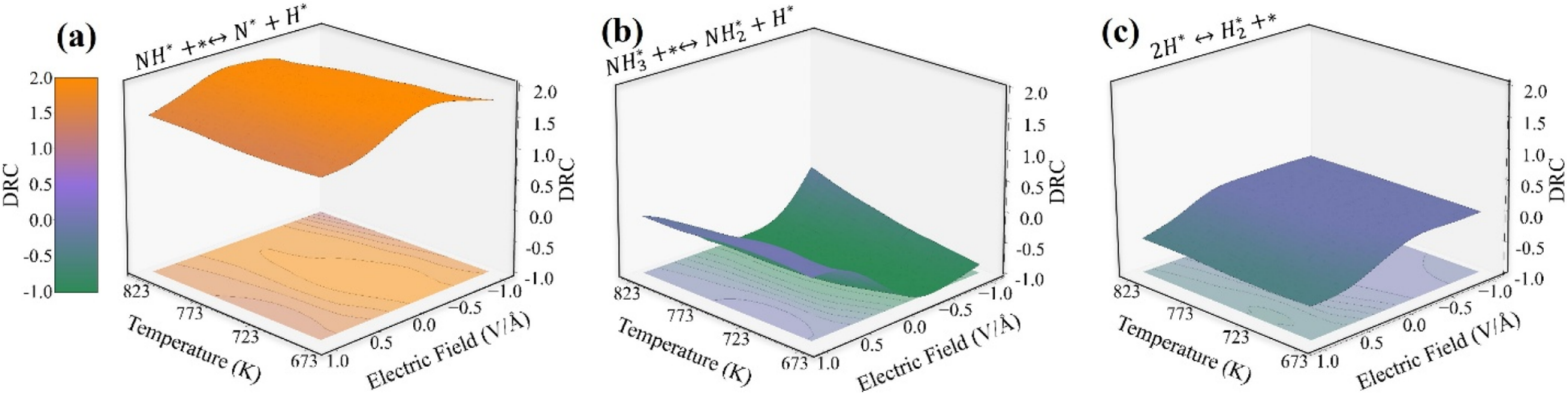
Temperature and electric field effects on the DRC of (a) NH dehydrogenation, (b) NH_3_ dehydrogenation, and (c) H_2_ formation during NH_3_ cracking on Ru(1013).

To further understand the electric field effect on the reaction mechanism of ammonia cracking over Ru(1013), we performed a detailed DRC analysis. Our findings reveal that, due to its relatively high activation energy (Figure 6 and Table S1), NH dehydrogenation (DRC > 1.0) remains the primary rate-limiting step (RLS) across all examined electric fields and temperatures (Figure 8a), consistent with NH_3_ cracking without the electric field. The surface coverage analysis of key intermediates under different conditions (Figure S39) shows that NH* remains the most abundant species, further validating the rate-limiting nature of NH dehydrogenation. The DRC analysis also found two key rate inhibitor reactions, NH_3_ dehydrogenation and H_2_ formation step in ammonia cracking on Ru(1013) under different electric fields. Under the negative fields, NH_3_ dehydrogenation acts as the only rate inhibitor step, with DRC values ranging from −1.0 to −0.2 (Figure 8b), whereas under the positive field region, we observe a competitive rate inhibition effect between NH_3_ dehydrogenation and H_2_ formation reaction (Figure 8c). For NH_3_ dehydrogenation, the inhibitory effect gradually diminishes with the DRC value increasing from −1 (0 V/Å) to 0 (+1 V/Å), while for H_2_ formation, the inhibition becomes stronger with the DRC value decreasing from 0 (0 V/Å) to −0.5 (+1 V/Å) across the examined temperature range (Figure S40).

To investigate the properties of these inhibitory reactions, we evaluated the coverage changes of free active site (θ*) and rate of NH dehydrogenation (RLS) over different electric fields by perturbing the rate constants of elementary steps. For the NH_3_ dehydrogenation, applying a −1 V/Å electric field and perturbing its rate constant by 1% consistently reduced the surface coverage of free active sites across all examined temperatures (Figure S41), and this leads to a lower rate of the rate-limiting step of NH dehydrogenation (Figure S42), thereby lowering the overall reaction rate. Under +1 V/Å, applying the same perturbation on the NH_3_ dehydrogenation step, the θ* remained nearly unchanged, confirming that this step does not inhibit NH_3_ cracking (Figure S43). Since H_2_ formation emerges as the competing rate-inhibiting step under positive electric fields (Figure 8c). The same perturbation of rate constant is performed on the H_2_ formation step under +1 V/Å, which confirms that the perturbation decreased the free active sites by ∼0.5% (Figure S43) and further reduced the rate of NH dehydrogenation by ∼0.5% examined across all examined temperatures (Figure S44), thereby confirming the shift of rate-inhibiting steps from NH_3_ dehydrogenation to H_2_ formation for ammonia cracking under +1 V/Å.

## 4 Conclusions

This study integrates DFT calculations and a microkinetic model to investigate NH_3_ cracking on Ru surfaces under various external electric fields. The DFT results reveal that the stepped Ru(1013) surface exhibits more favorable thermodynamic and kinetic energetics than Ru(0001) due to its low-coordinate B5 sites. The MKM results indicate that NH dehydrogenation is identified as the rate-limiting step, while NH_3_ dehydrogenation can act as a rate-inhibiting step by influencing active site availability and rate of rate-limiting step under the examined temperature of 673–823 K. By incorporating the electric field effects, we found that applying a negative electric field enhances NH_3_ cracking by accelerating the rate-limiting step of the NH dehydrogenation step, resulting in four orders of magnitude increase in TOF compared to the zero-field case, whereas positive electric fields inhibit the reaction. Bader charge analysis confirms that negative fields enrich electrons at the B5 sites to promote N–H bond dissociation. Overall, the field-driven MKM model offers a robust quantitative framework for optimizing field-enhanced catalysis, paving the way for the rational design of energy-efficient NH_3_ cracking systems based on fundamental principles rather than trial-and-error approaches.

## Supporting Information Available

Method details of DFT and MKM establishment; numerical and graphical analysis on energy diagrams; adsorption energy; reaction energy; activation barrier; MKM parameters; and surface coverages and degree of rate control (PDF)
All optimized geometries (CONTCARs) (ZIP)
