# Supplementary material for: Multiscale Simulation Guided Electric Field-Enhanced Ammonia Catalytic Cracking
Source: ACS Catal. 2025 Apr 24;15(10):7690–9. doi: 10.1021/acscatal.5c01829 (PMC12090187; doi:10.1021/acscatal.5c01829)
Supplement: Supplementary file 1 — cs5c01829_si_001.pdf [file cs5c01829_si_001.pdf]

Supporting Information

Multi-Scale Simulation Guided Electric Field-Enhanced Ammonia Catalytic Cracking

*Pragyansh Singh, Qiang Li, Yilang Liu, Fanglin Che**

Department of Chemical Engineering, University of Massachusetts Lowell, MA, 01854

*Corresponding Author Email: fanglin_che@uml.edu

S1. Computational Details

S1.1 Model Details

Geometry optimization calculations were performed to determine the most energetically favorable configurations of species participating in the ammonia decomposition reaction. We used two Ru surfaces to represent Ru catalysts: the thermodynamic most favorable Ru(0001) flat surface and the step Ru(1013) surface with the most favorable B5 site (**Figure S1**).[1] The Ru(0001) surface presents four distinct adsorption sites for species: top, bridge, hcp, and fcc. The Ru(1013) surface offers seven adsorption sites for reaction-relevant species: a hollow site between one upper top atom and two lower top atoms (h1), a hollow site between two upper top atoms and one lower top atom (h2), a four-fold hollow site (S4), upper top (Ut), lower top (Lt), upper bridge (Ub), lower bridge (Lb), and upper-lower bridge (ULb).

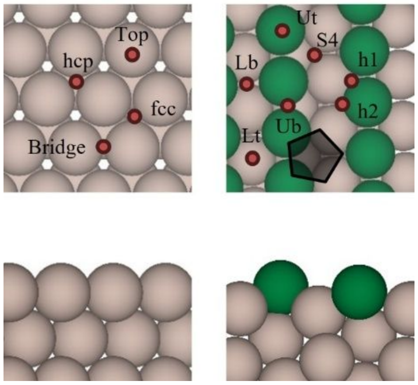

Figure S1. Top and side views of Ru(0001) and Ru (1013) catalytic surfaces. All possible adsorption sites over Ru(0001) and Ru(1013) surfaces are marked in red. Black pentagon area refers to B5 site. Color code: Beige for Ru and Green for Ru stepped atoms.

The potential adsorption configurations for the intermediates involved in the ammonia decomposition reaction on both Ru surfaces are presented in **Figures S2–S15**. To investigate the influence of the electric field on the energetics of these intermediates, a field range of -1.0 to +1.0 V/Å was applied, with a step interval of 0.2 V/Å. The adsorption energy was defined according to **Equation S1**.

$$E_{ad}(F) = E_{Total}(F) - E_{surface}(F) - E_{adsorbate} \qquad (S1)$$

The total energy of the species on the surface under the influence of the electric field is represented by $E_{Total}(F)$. The energy of the clean surface in the presence of the field is denoted

as $E_{surface}(F)$. The energy of the adsorbate without any field effect is represented by $E_{adsorbate}$. In the presence of uniform electric field (F), the field dependent adsorption energy (E_{ad}) can be expanded with Taylor series in the term of the electric field. A second order truncation of the Taylor series will result in **Equation S2**.[2, 3]

$$E_{ad}(F) = E_{ad}(0) - \Delta\vec{\mu}.\vec{F} - \frac{1}{2}\Delta\alpha\left|\vec{F}\right|^2 \qquad (S2)$$

where $\Delta\vec{\mu}$ and $\Delta\alpha$ are effective dipole moment and polarizability of the adsorbates on the surface,[4-6] and $E_{ad}(0)$ is the adsorption energy without any field.

Figures S16 and S17 present the field-dependent adsorption energies of the ammonia-cracking involved intermediates on Ru(1013) and Ru(0001) surfaces.

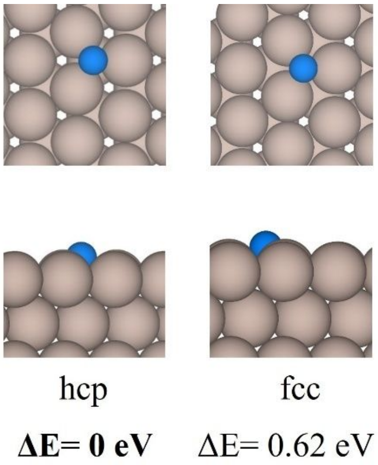

Figure S2. The possible adsorption configurations of N* over the Ru(0001) surface and its corresponding energetics. The most favorable adsorption geometry is when N* sits at the hcp site on the Ru(0001) surface. Color code: Blue for nitrogen.

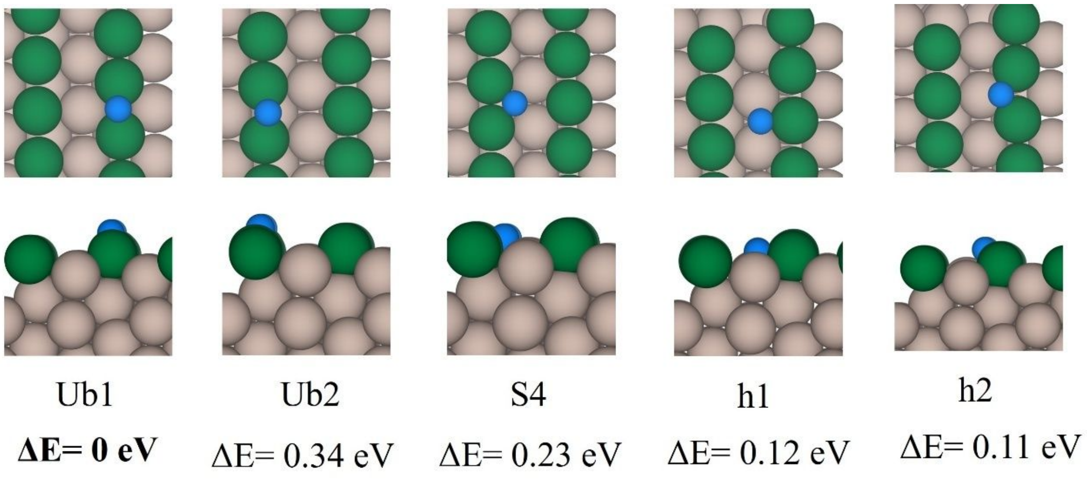

Figure S3. The possible adsorption configurations of N* over the Ru(1013) surface and its corresponding energetics. The most favorable adsorption geometry is when N* sits at the Ub1 site on the Ru(1013) surface.

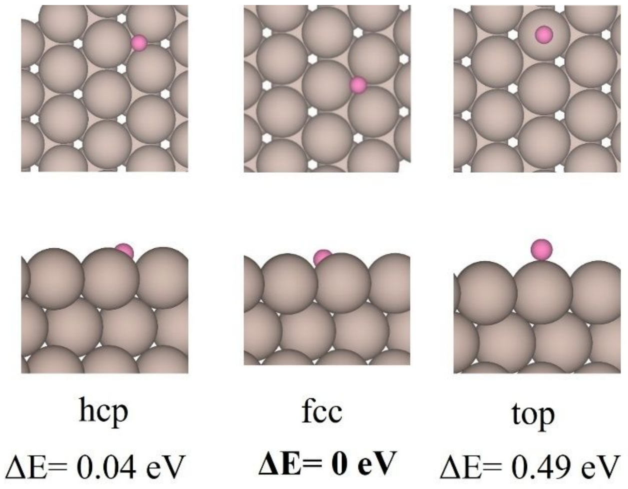

Figure S4. The possible adsorption configurations of H* over the Ru(0001) surface and its corresponding energetics. The most favorable adsorption geometry is when H* sits at the fcc site on the Ru(0001) surface. Color code: Pink for hydrogen.

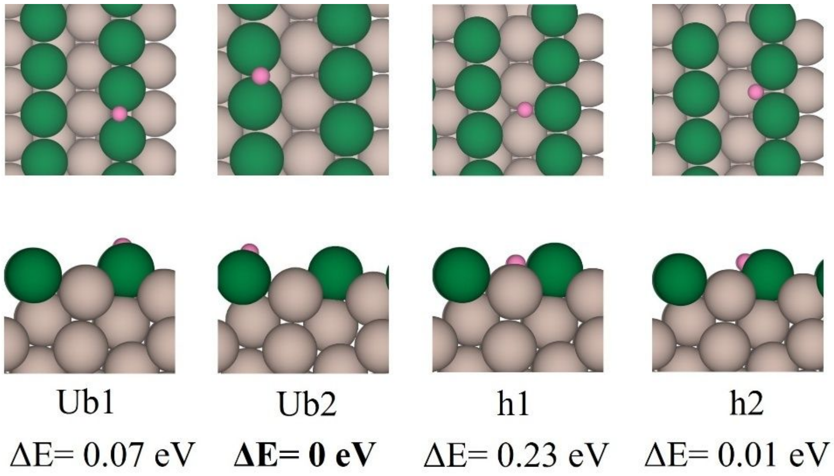

Figure S5. The possible adsorption configurations of H* over the Ru(1013) surface and its corresponding energetics. The most favorable adsorption geometry is when H* sits at the Ub2 site on the Ru(1013) surface.

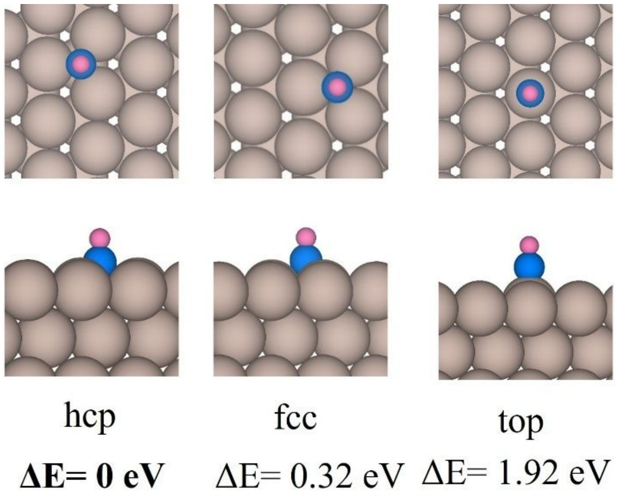

Figure S6. The possible adsorption configurations of NH* over the Ru(0001) surface and its corresponding energetics. The most favorable adsorption geometry is when NH* sits at the hcp site on the Ru(0001) surface.

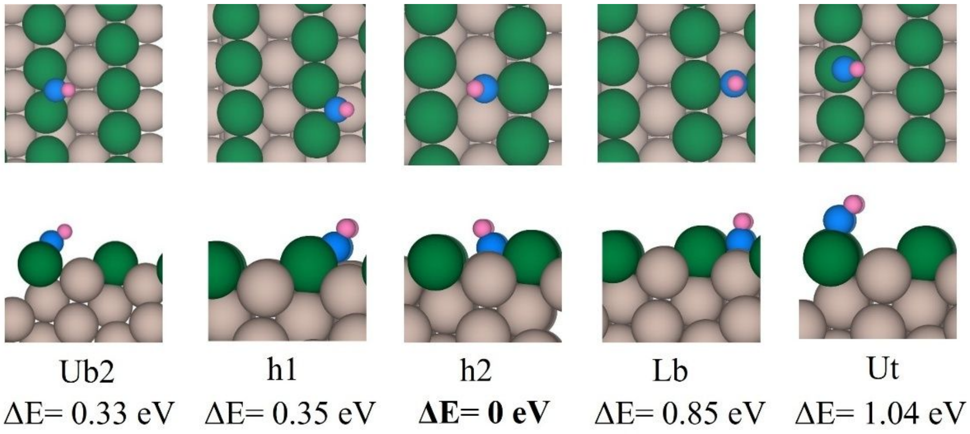

Figure S7. The possible adsorption configurations of NH* over the Ru(1013) surface and its corresponding energetics. The most favorable adsorption geometry is when NH* sits at the h2 site on the Ru(1013) surface.

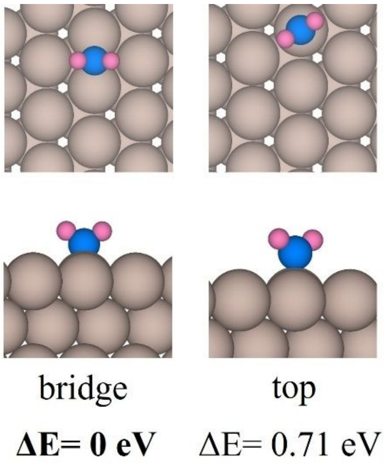

Figure S8. The possible adsorption configurations of NH_2* over the Ru(0001) surface and its corresponding energetics. The most favorable adsorption geometry is when NH_2* sits at the bridge site on the Ru(0001) surface.

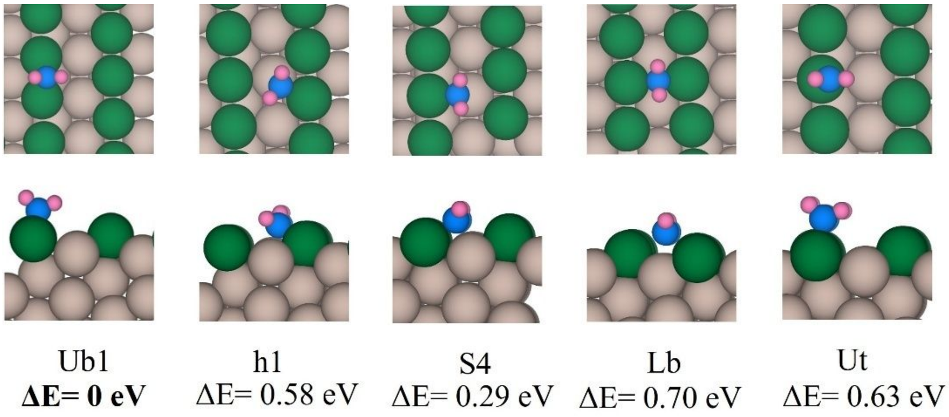

Figure S9. The possible adsorption configurations of NH_2* over the Ru(1013) surface and its corresponding energetics. The most favorable adsorption geometry is when NH_2* sits at the Ub1 site on the Ru(1013) surface.

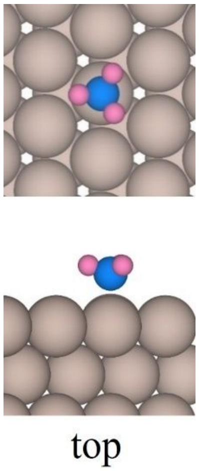

Figure S10. The possible adsorption configurations of NH_3* over the Ru(0001) surface and its corresponding energetics. The most favorable adsorption geometry is when NH_3* sits at the top site on the Ru(0001) surface.

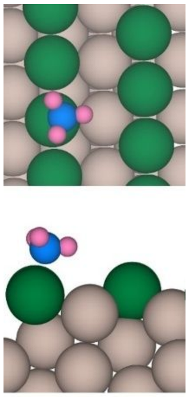

Figure S11. The possible adsorption configurations of NH_3* over the Ru(1013) surface and its corresponding energetics. The most favorable adsorption geometry is when NH_3* sits at the Ut site on the Ru(1013) surface.

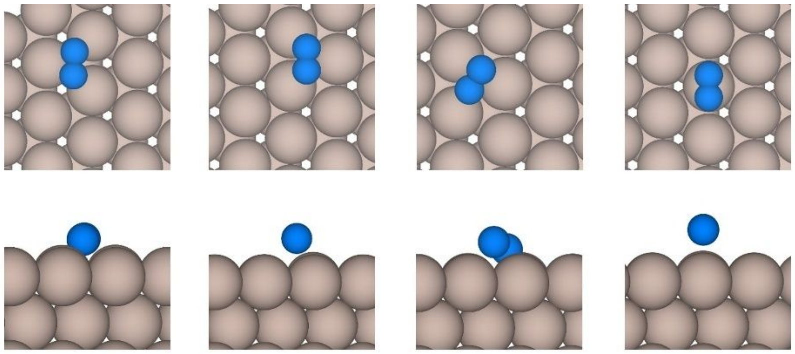

Figure S12. The possible adsorption configurations of N_2* over the Ru(0001) surface and its corresponding energetics. The most favorable adsorption geometry is when N_2* sits at the hcp site on the Ru(0001) surface.

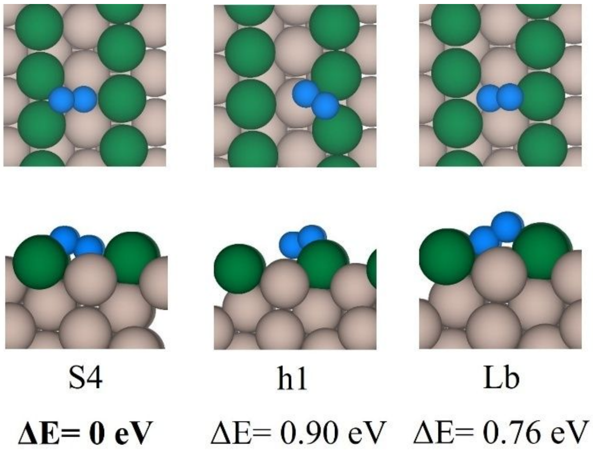

Figure S13. The possible adsorption configurations of N_2* over the Ru(1013) surface and its corresponding energetics. The most favorable adsorption geometry is when N_2* sits at the S4 site on the Ru(1013) surface.

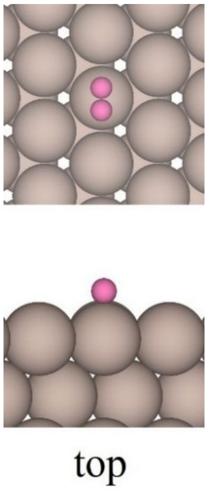

Figure S14. The possible adsorption configurations of H_2* over the Ru(0001) surface and its corresponding energetics. The most favorable adsorption geometry is when H_2* sits at the top site on the Ru(0001) surface.

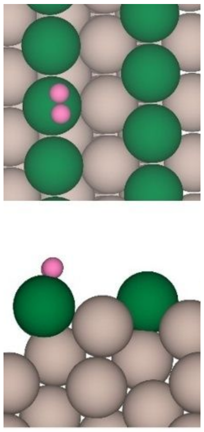

Ut

Figure S15. The possible adsorption configurations of H_2* over the Ru(1013) surface and its corresponding energetics. The most favorable adsorption geometry is when H_2* sits at the Ut site on the Ru(1013) surface.

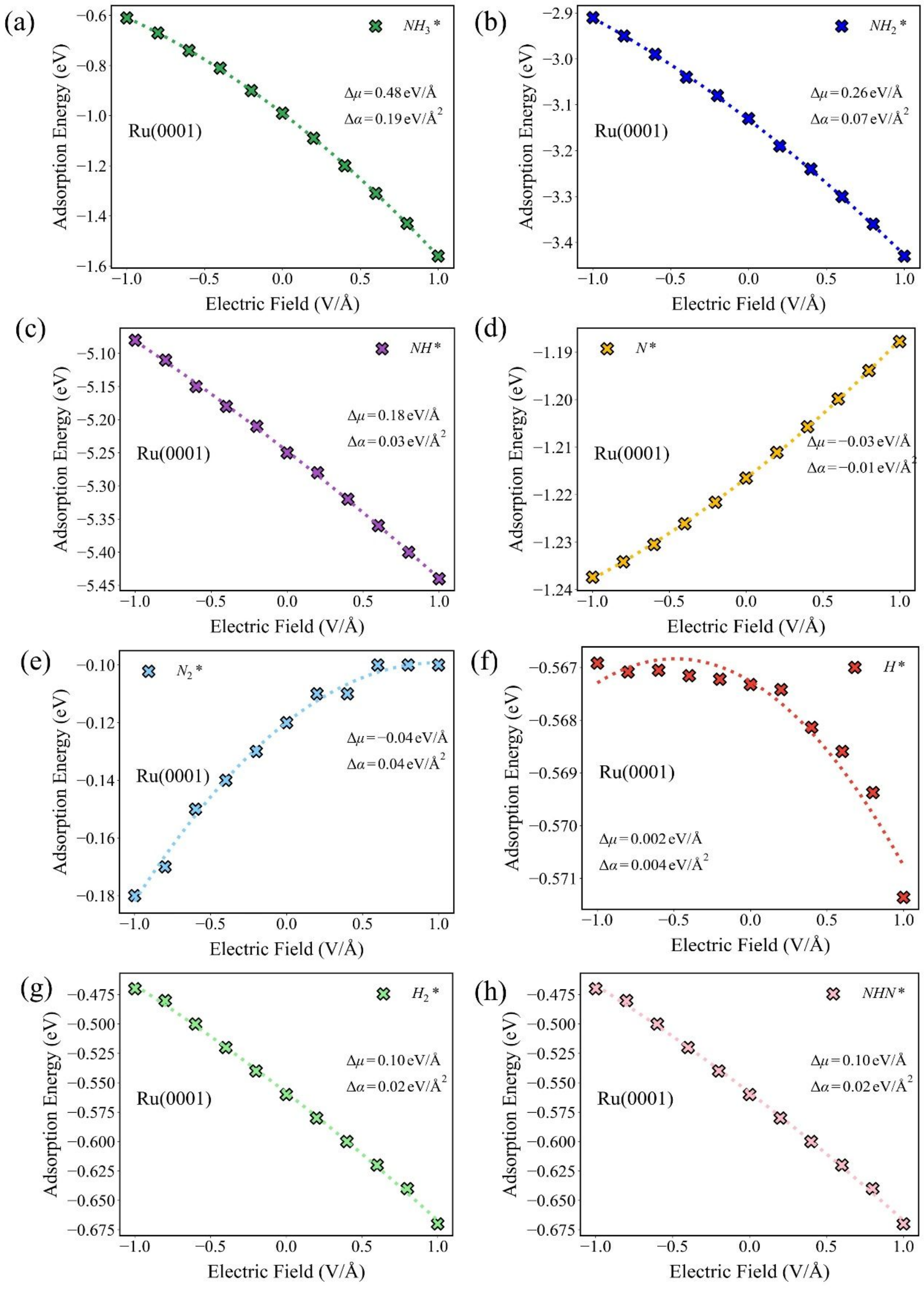

Figure S16. The electric fields effect on altering the adsorption energies of the most favorable adsorption configurations of (a) NH_3*, (b) NH_2*, (c) NH*, (d) N*, (e) N_2*, (f) H*, (g) H_2*, and (h) NHN* over Ru(0001).

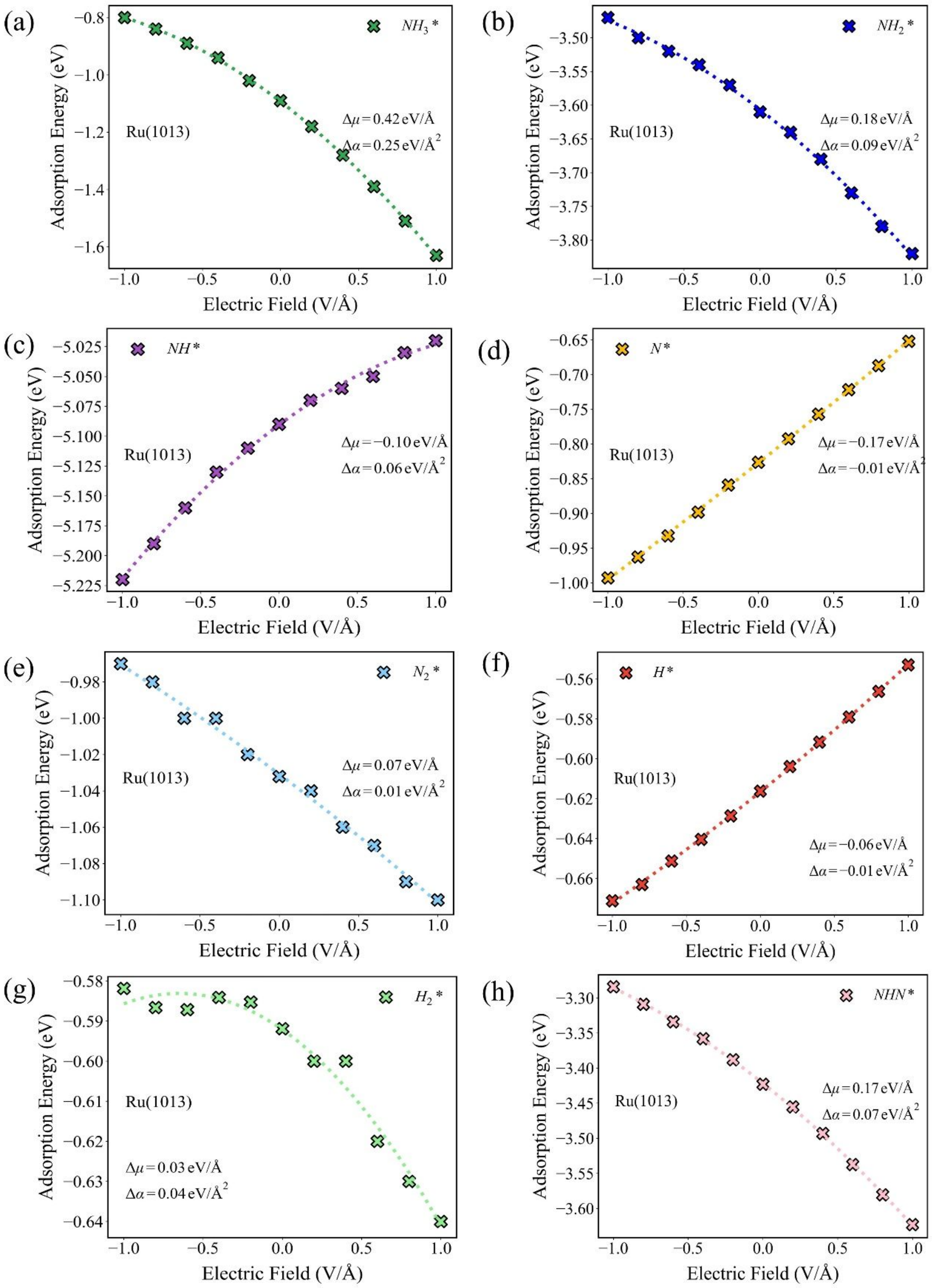

Figure S17. The electric fields effect on altering the adsorption energies of the most favorable adsorption configurations of (a) NH_3*, (b) NH_2*, (c) NH*, (d) N*, (e) N_2*, (f) H*, (g) H_2*, and (h) NHN* over Ru(1013).

The co-adsorption configurations of the intermediates were modelled on both the surfaces with the thermodynamically most stable geometries (**Figures S18–29**). Reaction energies were calculated (**Figures S30-S31**), as defined in **Equation S3**.

$$\Delta H_{rxn}(F) = E_{FS}(F) - E_{IS}(F) \qquad (S3)$$

where $E_{FS}(F)$ and $E_{IS}(F)$ are the DFT energies of the final state (FS) and initial state (IS) of each elementary step in the presence of electric field.

In the presence of uniform electric field (F), the field dependent reaction energy (ΔH_{rxn}) can be expanded with Taylor series in terms of the electric fields. A second order truncation of the Taylor series will result in **Equation S4**.[7-9]

$$\Delta H_{rxn}(F) = \Delta H_{rxn}(0) - \Delta\vec{\mu}_{rxn}.\vec{F} - \frac{1}{2}\Delta\alpha_{rxn}\left|\vec{F}\right|^2 \qquad (S4)$$

where $\Delta\vec{\mu}_{rxn}$ and $\Delta\alpha_{rxn}$ are the changes in the effective dipole moment and polarizability between final and initial state of an elementary reaction. While $\Delta H_{rxn}(0)$ is the reaction energy of the elementary step without any field.

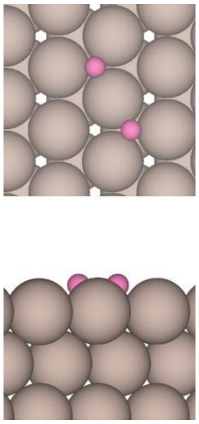

Figure S18. The co-adsorption configuration of 2H* over the Ru(0001) surface.

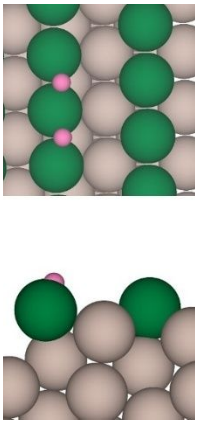

Figure S19. The co-adsorption configuration of 2H* over the Ru(1013) surface.

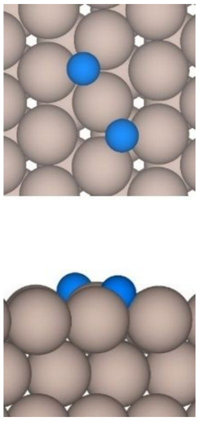

Figure S20. The co-adsorption configuration of 2N* over the Ru(0001) surface.

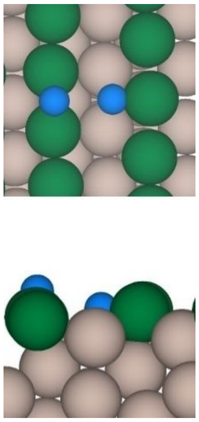

Figure S21. The co-adsorption configuration of 2N* over the Ru(1013) surface.

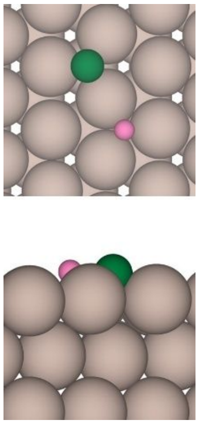

Figure S22. The co-adsorption configuration of N* and H* over the Ru(0001) surface.

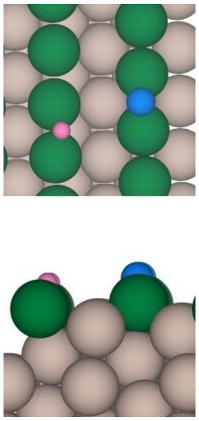

Figure S23. The co-adsorption configuration of N* and H* over the Ru(1013) surface.

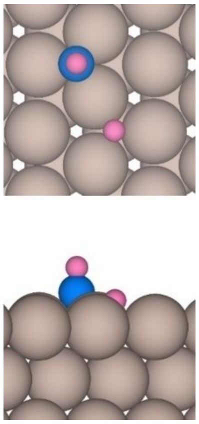

Figure S24. The co-adsorption configuration of NH* and H* over the Ru(0001) surface.

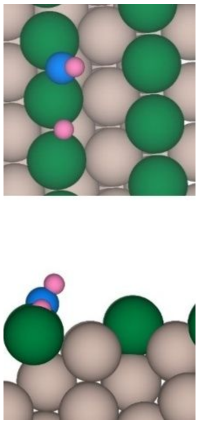

Figure S25. The co-adsorption configuration of NH* and H* over the Ru(1013) surface.

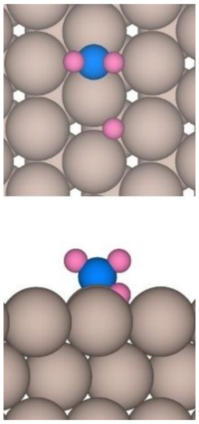

Figure S26. The co-adsorption configuration of NH_2* and H* over the Ru(0001) surface.

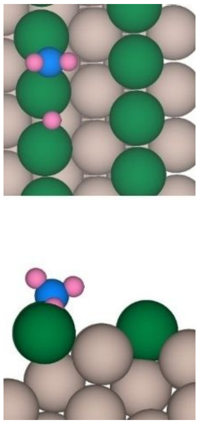

Figure S27. The co-adsorption configuration of NH_2* and H* over the Ru(1013) surface.

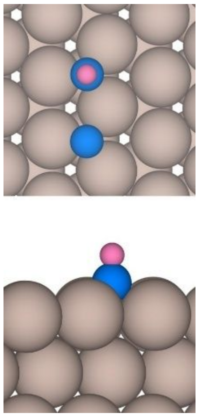

Figure S28. The co-adsorption configuration of NH* and N* over the Ru(0001) surface.

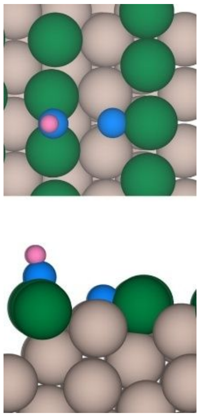

Figure S29. The co-adsorption configuration of NH* and H* over the Ru(1013) surface.

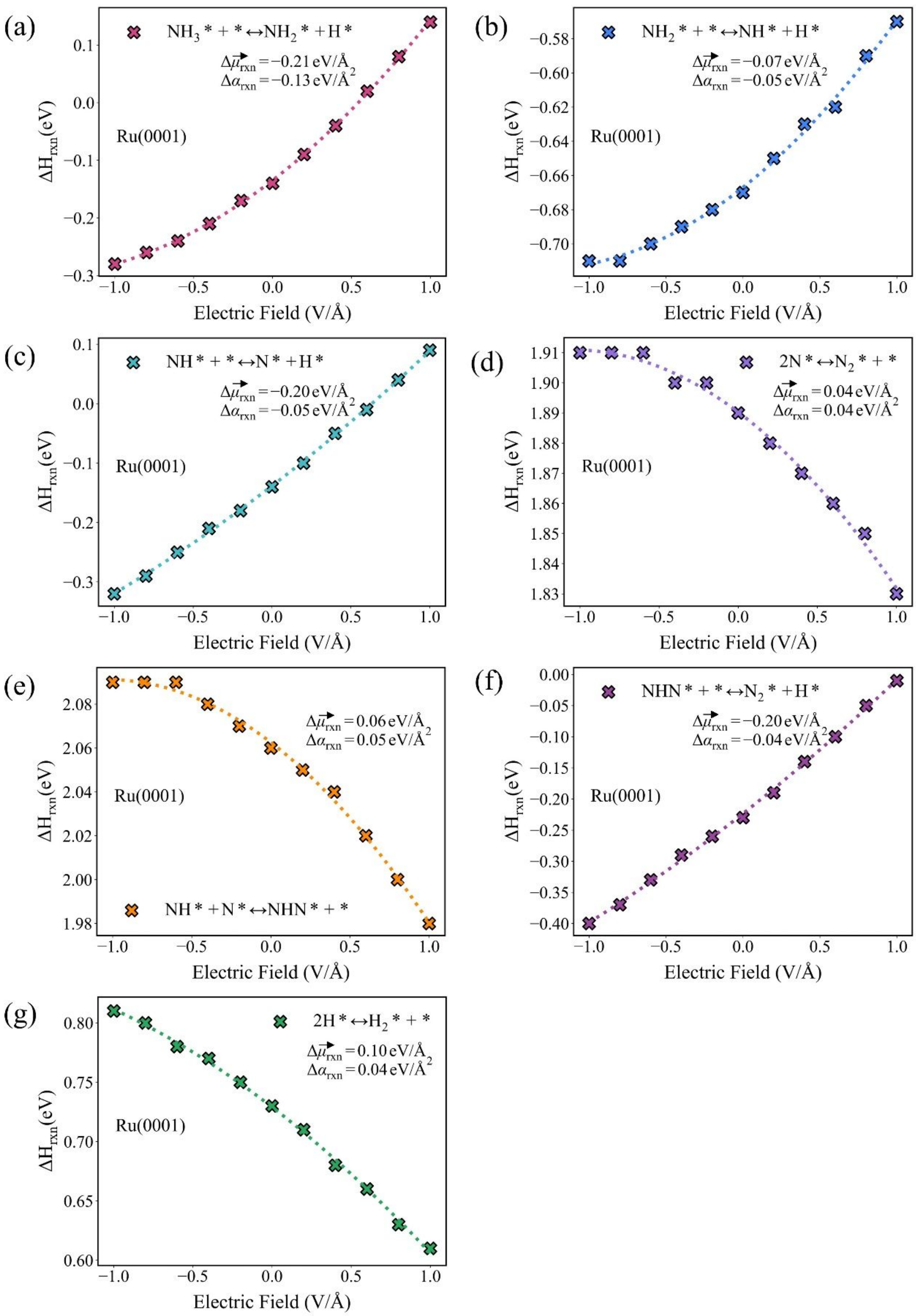

Figure S30. The electric fields effect on altering the reaction energies of the elementary steps involved in the ammonia cracking over Ru(0001) surface.

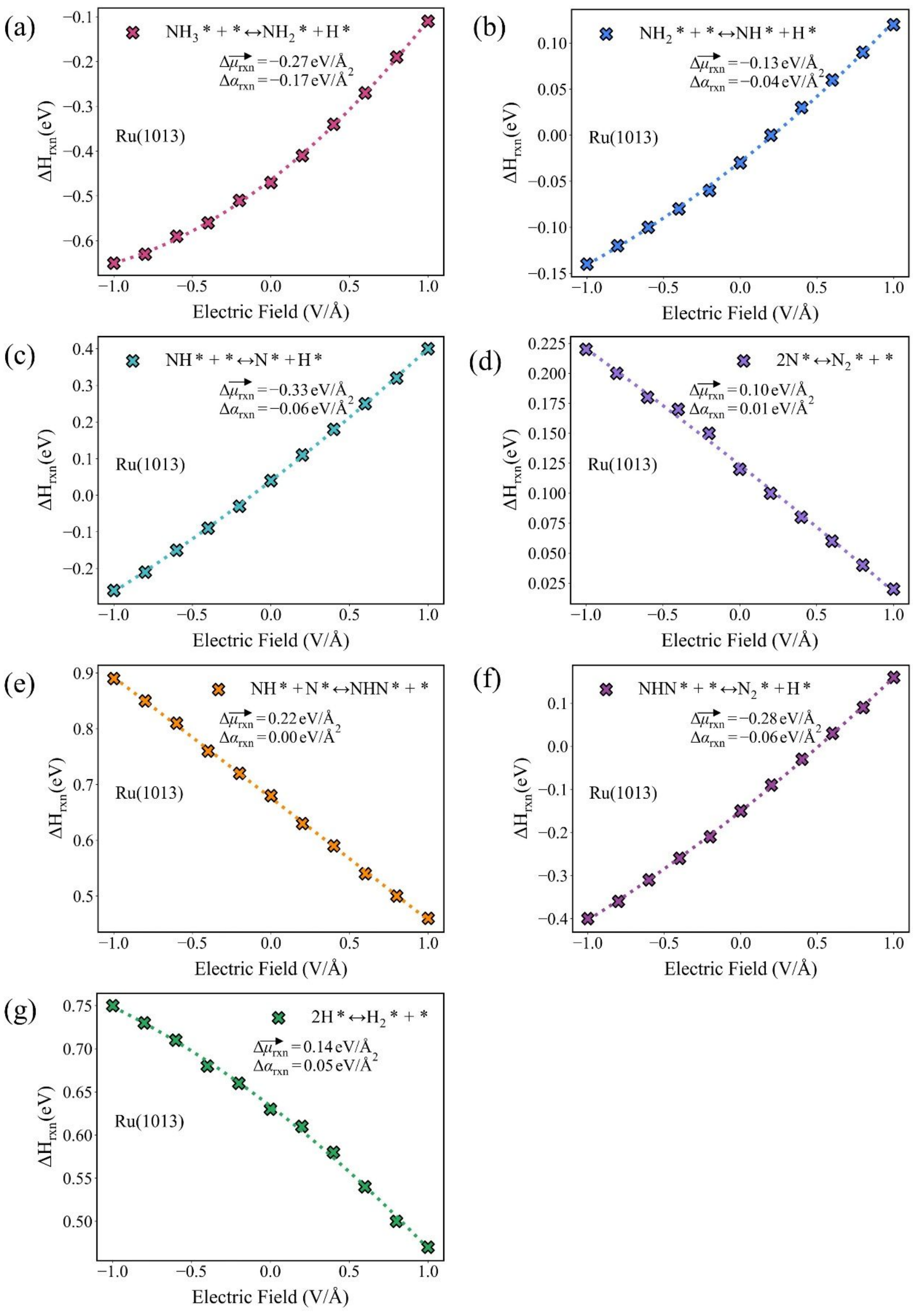

Figure S31. The electric fields effect on altering the reaction energies of the elementary steps involved in the ammonia cracking over Ru(1013) surface.

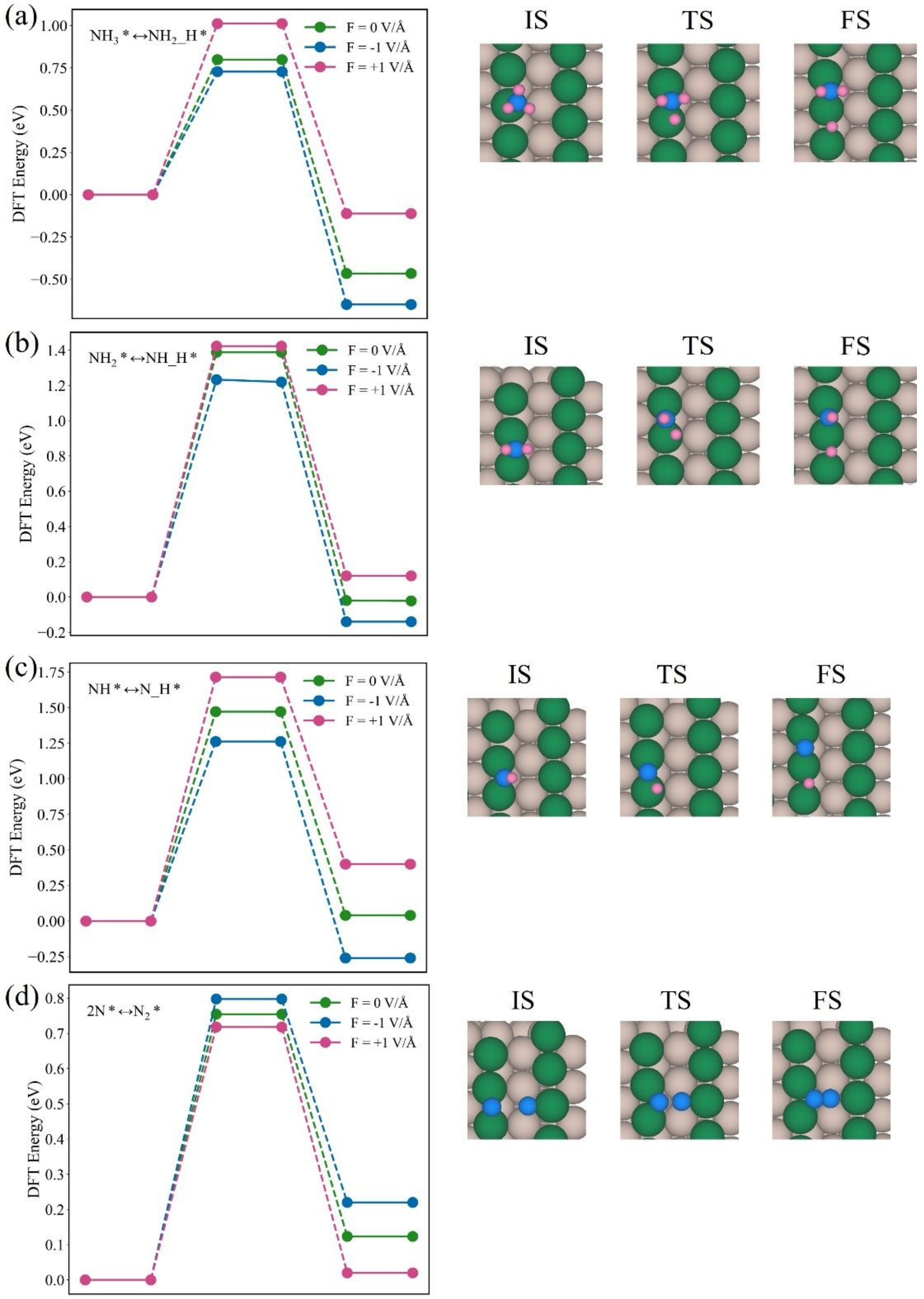

Figure S32. Field dependent activation energy of: (a) NH_3 dehydrogenation step, (b) NH_2 dehydrogenation step, (c) NH dehydrogenation step, and (d) N_2 formation step with the corresponding geometries of initial state (IS), transition state (TS), and final state (FS).

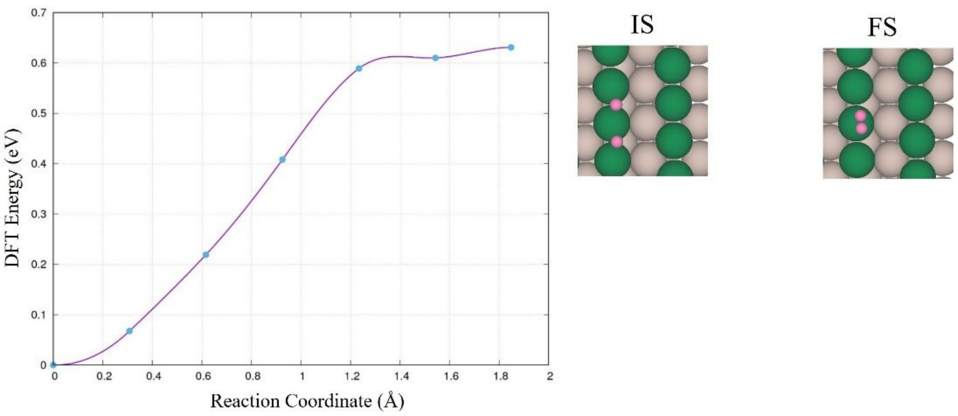

Figure S33. Simulated minimum energy path (MEP) of H_2 formation step by CI-NEB. The MEP reflects the absence of saddle point. The corresponding geometries of initial and final state are mentioned as IS and FS respectively.

The field-dependent activation energy (E_a) can be expanded with a second order Taylor series in terms of the electric fields in **Equation S5**:

$$E_a(F) = E_a(0) - \Delta\vec{\mu}_a.\vec{F} - \frac{1}{2}\Delta\alpha_a\left|\vec{F}\right|^2 \qquad (S5)$$

where $\Delta\vec{\mu}_a$ and $\Delta\alpha_a$ are the changes in the effective dipole moment and polarizability between transition state and initial state of an elementary reaction (**Figure S34**). While $E_a(0)$ is the activation barrier of the elementary step without any fields.

Comparison between the changes in effective dipole moments in reaction energetics ($\Delta\vec{\mu}_{rxn}$ in **Figures 31a-d**) and in activation barriers ($\Delta\vec{\mu}_a$ in **Figures S34a-d**), shows that although they have the same sign for each elementary step, $\Delta\vec{\mu}_{rxn}$ consistently has a greater magnitude. This suggests that the applied electric fields influence reaction energies more strongly than activation energies across these steps. This observation aligns with the BEP correlations, where the slope is consistently less than 1, indicating that electric fields have a greater impact on reaction energetics than on activation barriers.

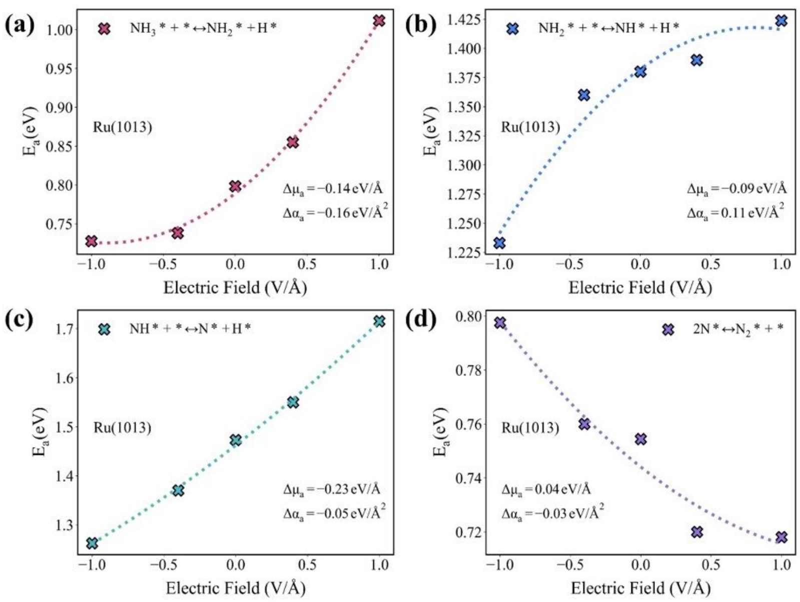

Figure S34. The electric fields effect on altering the activation energies of the elementary steps involved in the ammonia cracking over Ru(1013) surface.

We conducted Bader charge calculations for the flat Ru(0001) surface, as shown in **Figure S35**. The comparative analysis reveals that the charge distribution on Ru(0001) is uniform, whereas the Ru(1013) surface shows heterogeneous charge distribution at the kinked B5 sites (**Figures 5**). Furthermore, under a negative electric field of –1 V/Å, the B5 sites on the Ru(1013) surface accumulate more negative charge than those on the flat Ru(0001) surface. This enhanced charge accumulation at the B5 sites likely contributes to the greater catalytic activity of Ru(1013) in promoting ammonia decomposition compared to Ru(0001) under negative electric fields (**Figures S30-31**).

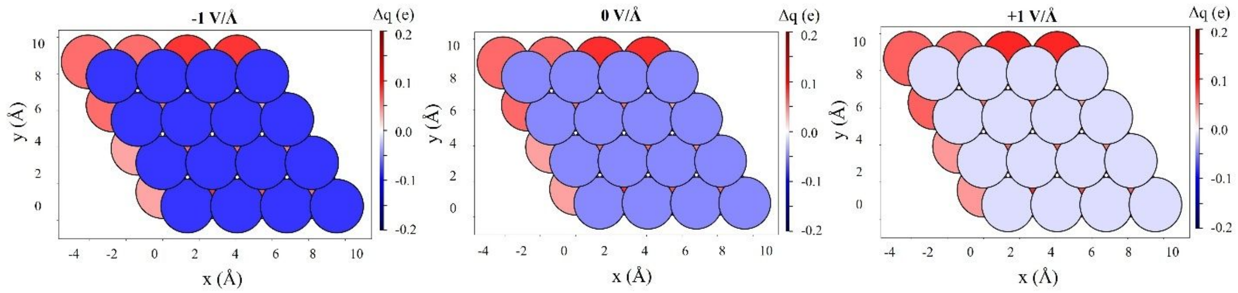

Figure S35. Top view of Bader charge distribution on the Ru(0001) surface, under the applied electric field condition of (a) -1 V/Å, (b) 0 V/Å, and (c) +1 V/Å. The mentioned Δq represents the net charge on the atom. A positive value indicates a positively charged atom, and vice versa.

We also investigated the sensitivity of catalytic reactions to electric fields—using the first N–H bond cleavage as a representative step—on the same catalyst (Ru) with different surface geometries, specifically the flat Ru(0001) and the kinked Ru(1013) surfaces. Our results show that the reaction on Ru(1013) is more strongly affected by the electric field due to its larger dipole moment ($\Delta\vec{\mu}_{rxn}$) and polarizability ($\Delta\alpha_{rxn}$), as illustrated in **Figures S36a and S36b**. To further isolate the effect of surface geometry, we examined the same (1013) surface structure on different metals, selecting Co and Re for their similar HCP crystal structures to Ru. Our DFT calculations indicate that for the same geometry and reaction, $\Delta\vec{\mu}_{rxn}$ and $\Delta\alpha_{rxn}$ are nearly identical across these metals, suggesting that field sensitivity is primarily governed by the surface geometry rather than the specific metal element (**Figures S36c and S36d**).

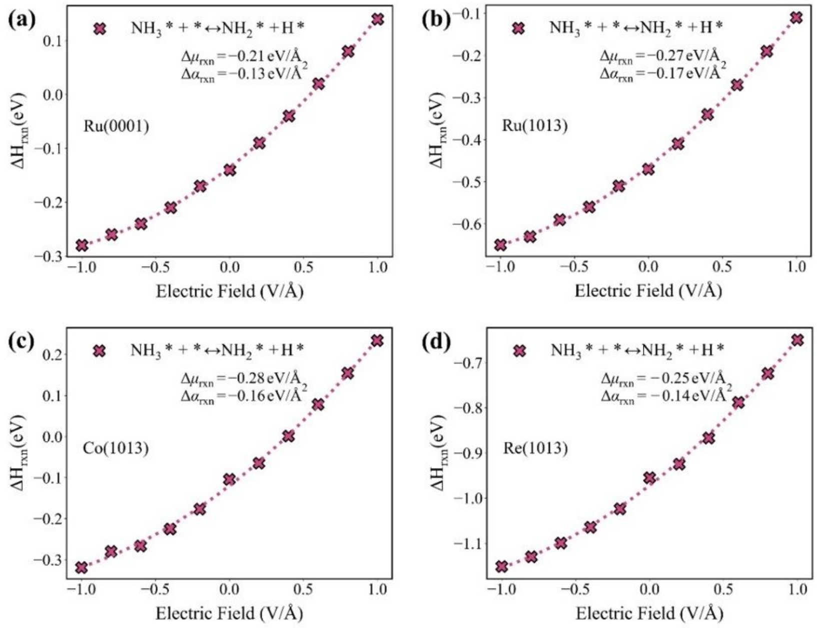

Figure S36. Field-dependent reaction energies of the first N-H bond in NH_3 over (a) Ru(0001), (b) Ru(1013), (c) Co(1013), and (d) Re(1013).

Table S1. DFT-calculated field-dependent activation energy (eV) over Ru(1013) surface .

Elementary Steps	Activation energy (eV)				
Electric Field (V/Å)	+1	+0.4	0	-0.4	-1
NH_3*+*↔NH_2*+H*	1.01	0.85	0.79	0.74	0.73
NH_2*+*↔NH*+H*	1.42	1.38	1.38	1.36	1.23
NH*+*↔N*+H*	1.71	1.55	1.47	1.37	1.26
2N*↔N_2*+*	0.71	0.72	0.75	0.76	0.79

S1.2 Microkinetic Model Setup

S1.2.1 Stoichiometric Consistency

Establishing the kinetic model requires defining the stoichiometric number, σ, for each elementary step. The eight elementary steps and the corresponding stoichiometric numbers involved in the most stable thermodynamic pathway for the ammonia cracking are presented in **Table S3.**

Table S3. Examined elementary steps and the corresponding stoichiometric number for the ammonia cracking microkinetic model.

Step Number	Elementary Steps	Stoichiometric Number (σ)
1.	NH_3(g)+* ↔NH_3*	2
2.	NH_3*+*↔NH_2*+H*	2
3.	NH_2*+*↔NH*+H*	2
4.	NH*+*↔N*+H*	2
5.	2N*↔N_2*+*	1
6.	N_2*↔N_2(g)+*	1
7.	2H*↔H_2*+*	3
8.	H_2*↔H_2(g)+*	3
Overall Reaction	2NH_3(g)↔N_2(g)+3H_2(g)	

S1.2.2 Micro-kinetic model parameters

In total, a system of eight differential equations were solved iteratively with Cantera python package to determine the steady-state solution. Additional input parameters for the microkinetic model are presented in **Table S4**.

Table S4. Micro-kinetic model input parameters.

Input parameters	Values
Pressure (p0)	1 atm
Volumetric velocity (v0)	30 cm^3/min
Feed composition	100 % NH_3 (g)
Ruthenium Density[10]	12.37 g/cm^3
Specific Area[10]	(30 to 50) E4 cm^2/g
Surface Density	1.83 E4 mol/cm^2
Catalyst area/volume (abyv)	4.94 E6 (cm^{-1})
Temperature range	673 – 823 K
Electric field range	-1 to +1 V/Å

Temperature, pressure, and feed composition values were taken from experiments.[11] Site density was calculated based on the dimensions of the Ru(1013) model, as shown below,

$$Surface\ Density = \frac{1}{Catalyst\ Area * N_A} = \frac{mol/cm^2}{90.34 * 10^{-16} * 6.022 * 10^{23}} = 1.83E4\ mol/cm^2$$

To calculate the abyv (Catalyst area/volume) parameter, the bulk density and specific area information of a typical catalyst is based on experiment data[10],

$$abyv = Density\left(\frac{gm}{cm^3}\right) * Specific\ area\left(\frac{cm^2}{gm}\right) = 12.37 * 40 * 10^4 = 4.94E6\ cm^{-1}$$

All MKM results were obtained at a consistent conversion, 0.1%. The modeling approach employed a gradient descent optimization method to determine the plug flow reactor (PFR) length required to achieve the target conversion of 0.1% (**Equations S6-S9**). The central difference method, within a small step value range (h), was used to calculate the gradient of conversion with respect to reactor length (**Equation S9**). Given that conversion is a function of reactor length (assuming all other variables remain constant), the algorithm was designed to minimize the loss function value relative to the target conversion (**Equation S7**). Consequently, the loss gradient with respect to the length parameter is defined by **Equation S8**. The length parameter was iteratively updated in each step, based on information derived from the gradient calculation.

$$L_{new} = L_{now} - \alpha * grad \tag{S6}$$

$$loss = (X - X_{target})^2 \tag{S7}$$

$$grad = \frac{d(loss)}{dL} = 2(X - X_{target})\frac{dX}{dL} \tag{S8}$$

$$\frac{dX}{dL} = \frac{X(L+h) - X(L-h)}{2h} \tag{S9}$$

A gradient descent algorithm was employed to minimize the loss function, defined using the squared error. To enhance model flexibility and adaptability, an Adam optimizer was integrated, enabling dynamic adjustment of the learning rate (α) hyperparameter during each iteration of reactor length (L) updates. Upon determination of the optimal reactor length (L_{opt}), the turnover frequency (TOF) was calculated. The TOF (s^{-1}) is defined as a function of the target conversion and residence time (**Equation S10**):

$$TOF = \frac{C_{NH3,in(g)} - C_{NH3,out(g)}}{\tau * C_{NH3,in(s)} * catalyst(\frac{area}{volume})} \tag{S10}$$

Given the assumption of uniform reaction across the reactor's cross-sectional area, calculations were simplified by utilizing reactor length instead of volume. Similarly, Length velocity was used instead of volumetric velocity. This simplification is justified by the uniformity

assumption and facilitates a more straightforward analysis of reactor performance. Consequently, the residence time (τ) can be expressed as (**Equation S11**):

$$\tau = \frac{L_{reactor}}{v_o} \qquad (S11)$$

S1.2.3 Degree of rate control and Degree of thermodynamic rate control

The kinetic importance of steps in the reaction mechanism can be evaluated with the degree of rate control calculation[12, 13] in the MKM by implementing the **Equation S12**.

$$X_{RC,i} = \frac{k_i}{r}\left(\frac{dr}{dk_i}\right) = \frac{dlnr}{dlnk_i} = \left(\frac{dlnr}{d(\frac{-G_i^{\ddagger}}{RT})}\right) \qquad (S12)$$

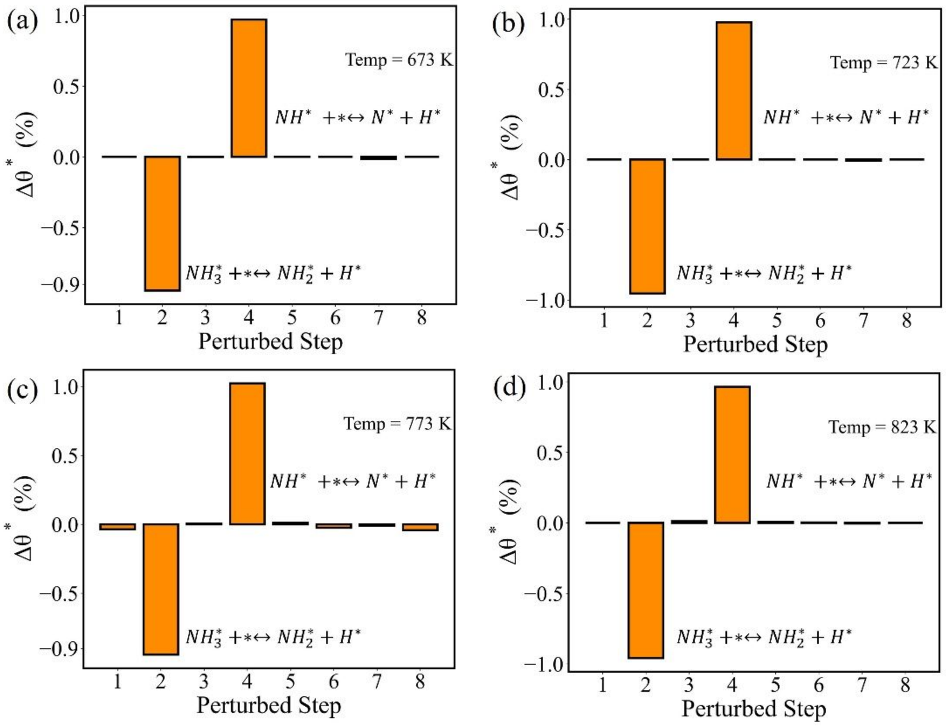

Figure S37. The impact of a 1% perturbation in the rate constant of each elementary step on the percentage change in surface coverage of free active sites across the examined temperatures: (a)

673 K, (b) 723 K, (c) 773 K, and (d) 823 K. The corresponding elementary steps numbered in the graph are: (1) NH_3(g)+*↔NH_3*, (2) NH_3*+*↔NH_2*+H*, (3) NH_2*+*↔NH*+H*, (4) NH*+*↔N*+H*, (5) 2N*↔N_2*+*, (6) N_2*↔N_2(g)+*, (7) 2H*↔H_2*+*, (8) H_2*↔H_2(g)+*. It is observed that across all the examined temperature points, perturbing the rate constant of NH_3 dehydrogenation step leads to decrease in free active site whereas opposite is observed for NH dehydrogenation step.

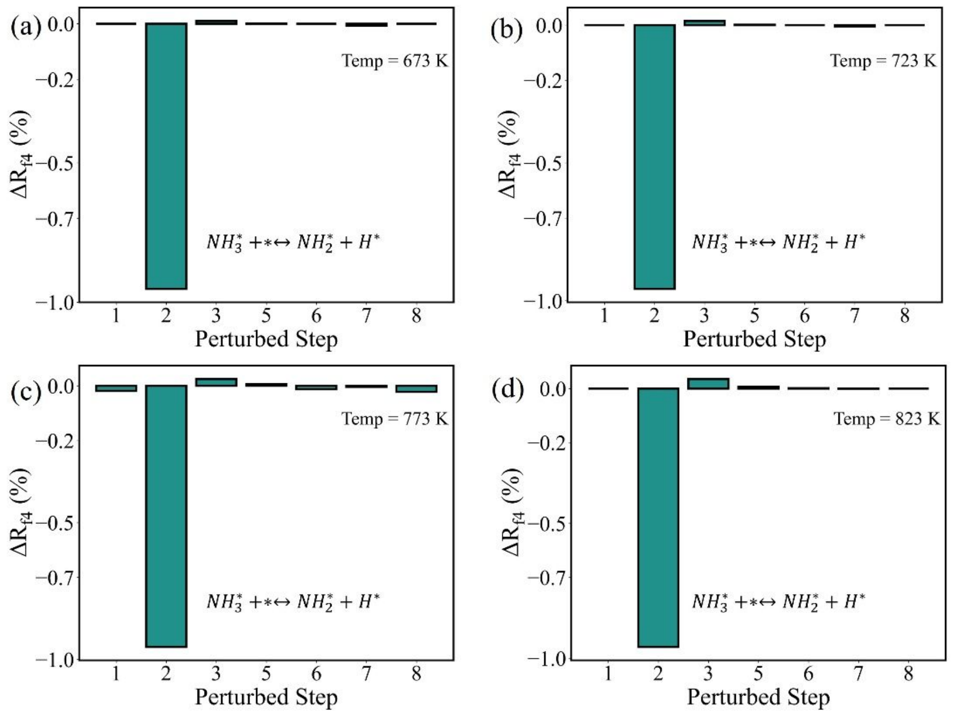

Figure S38. The impact of a 1% perturbation in the rate constant of each elementary step on the percentage change in the forward rate of the NH dehydrogenation step (Rf4) across the examined temperatures: (a) 673 K, (b) 723 K, (c) 773 K, and (d) 823 K. The corresponding elementary steps numbered in the graph are: (1) NH_3(g)+*↔NH_3*, (2) NH_3*+*↔NH_2*+H*, (3) NH_2*+*↔NH*+H*, (5) 2N*↔N_2*+*, (6) N_2*↔N_2(g)+*, (7) 2H*↔H_2*+*, (8) H_2*↔H_2(g)+*. The graph skips plotting the (4) NH*+*↔N*+H* step bar data, as it is intuitive that increasing the rate constant of a step will obviously result into enhancement the rate of that reaction. It is observed that across all the examined temperature points, perturbing the rate constant of NH_3 dehydrogenation step leads to reduction in rate of rate-limiting step (R_{f4}).

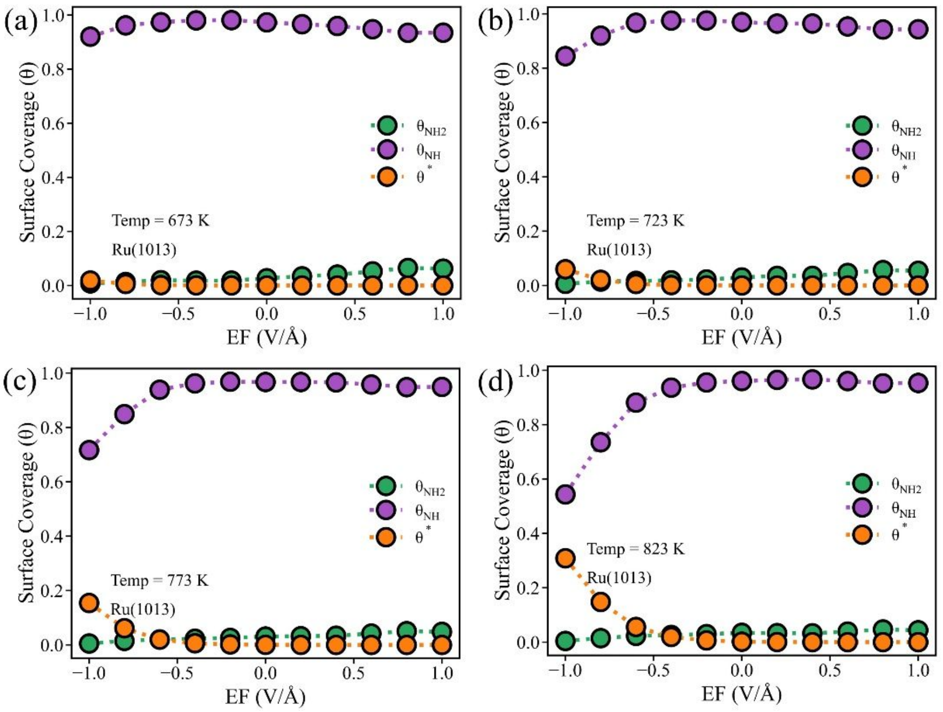

Figure S39. Field-dependent surface coverage of relevant intermediate species at examined temperatures of (a) 673 K, (b) 723 K, (c) 773 K, and (d) 823 K.

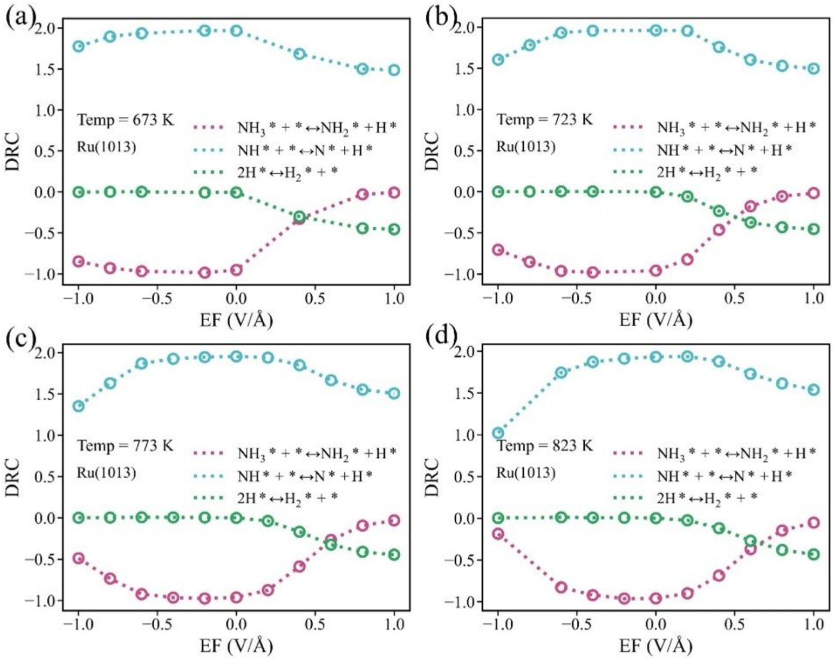

Figure S40. Field-dependent degree of rate control (DRC) values of key elementary steps over Ru(1013) at the examined temperatures of (a) 673 K, (b) 723 K, (c) 773 K, and (d) 823 K.

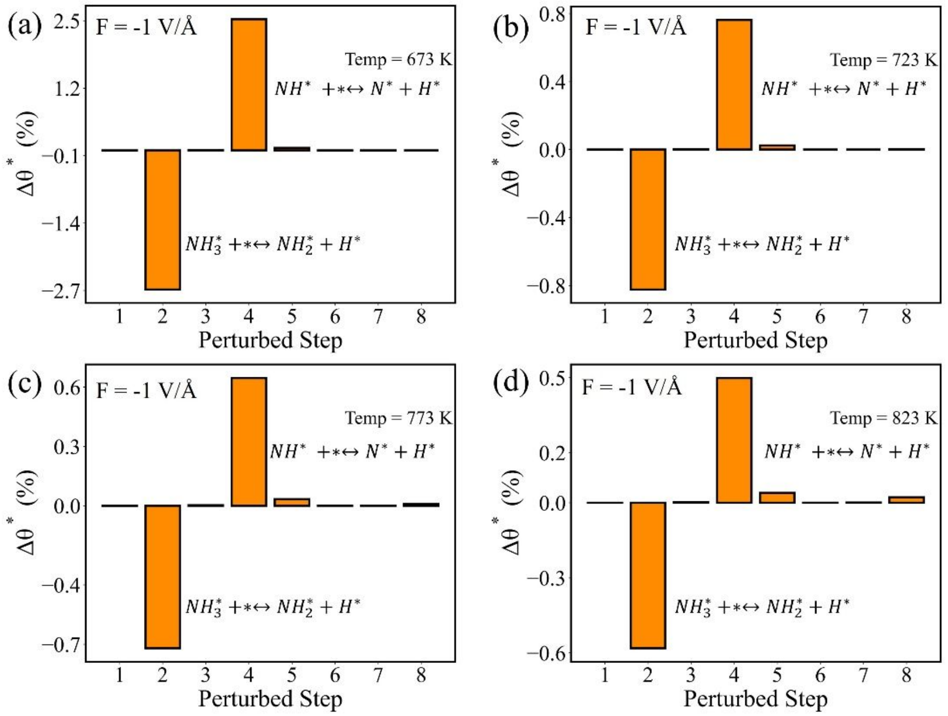

Figure S41. At the applied electric field of -1 V/Å, The impact of a 1% perturbation in the rate constant of each elementary step on the percentage change in surface coverage of free active sites across the examined temperatures: (a) 673 K, (b) 723 K, (c) 773 K, and (d) 823 K. The corresponding elementary steps numbered in the graph are: (1) $NH_3(g)+*\leftrightarrow NH_3*$, (2) $NH_3*+*\leftrightarrow NH_2*+H*$, (3) $NH_2*+*\leftrightarrow NH*+H*$, (4) $NH*+*\leftrightarrow N*+H*$, (5) $2N*\leftrightarrow N_2*+*$, (6) $N_2*\leftrightarrow N_2(g)+*$, (7) $2H*\leftrightarrow H_2*+*$, (8) $H_2*\leftrightarrow H_2(g)+*$. It is observed that across all the examined temperature points, perturbing the rate constant of NH_3 dehydrogenation step leads to decrease in free active site whereas opposite is observed for NH dehydrogenation step.

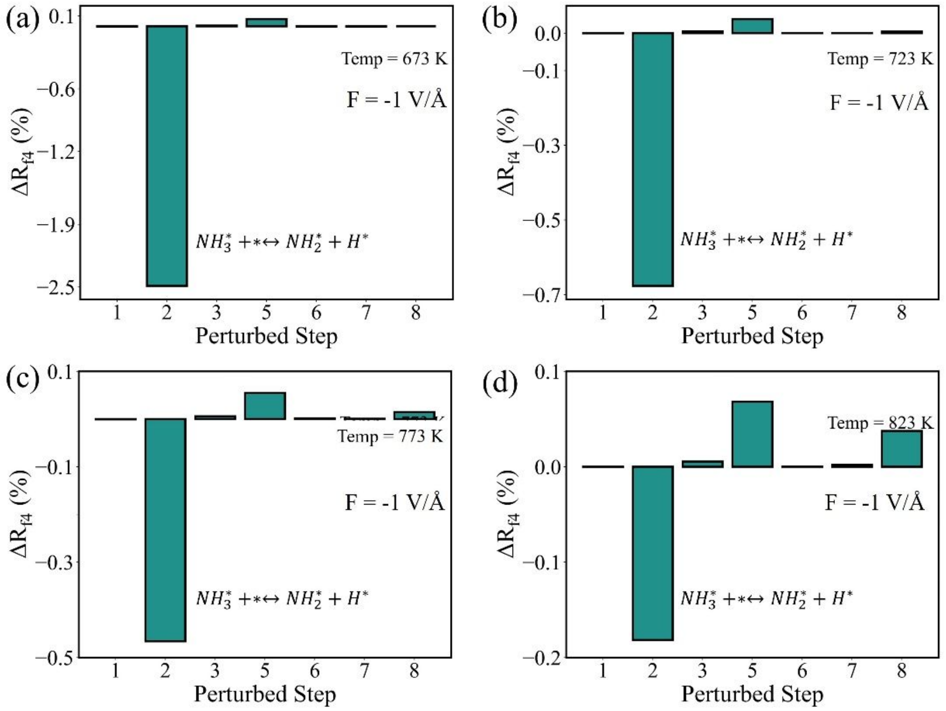

Figure S42. At the applied electric field of -1 V/Å, The impact of a 1% perturbation in the rate constant of each elementary step on the percentage change in the forward rate of the NH dehydrogenation step (Rf4) across the examined temperatures: (a) 673 K, (b) 723 K, (c) 773 K, and (d) 823 K. The corresponding elementary steps numbered in the graph are: (1) $NH_3(g)+*\leftrightarrow NH_3*$, (2) $NH_3*+*\leftrightarrow NH_2*+H*$, (3) $NH_2*+*\leftrightarrow NH*+H*$, (5) $2N*\leftrightarrow N_2*+*$, (6) $N_2*\leftrightarrow N_2(g)+*$, (7) $2H*\leftrightarrow H_2*+*$, (8) $H_2*\leftrightarrow H_2(g)+*$. The graph skips plotting the (4) $NH*+*\leftrightarrow N*+H*$ step bar data, as it is intuitive that increasing the rate constant of a step will obviously result into enhancement the rate of that reaction. It is observed that across all the examined temperature points, perturbing the rate constant of NH_3 dehydrogenation step leads to reduction in rate of rate-limiting step (R_{f4}).

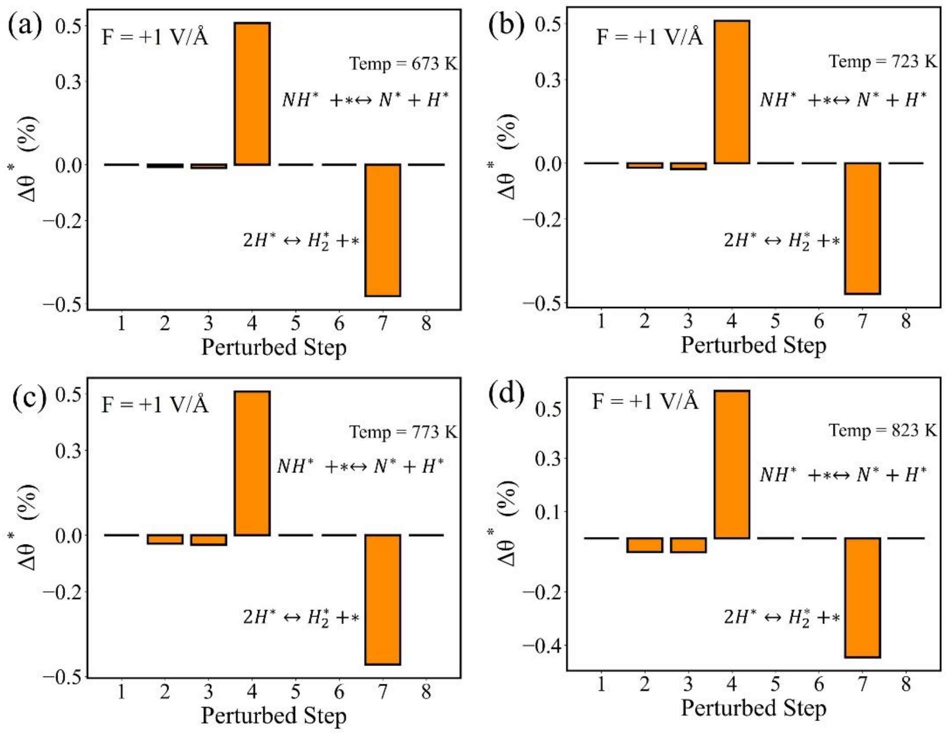

Figure S43. At the applied electric field of +1 V/Å, The impact of a 1% perturbation in the rate constant of each elementary step on the percentage change in surface coverage of free active sites across the examined temperatures: (a) 673 K, (b) 723 K, (c) 773 K, and (d) 823 K. The corresponding elementary steps numbered in the graph are: (1) $NH_3(g)+*\leftrightarrow NH_3*$, (2) $NH_3*+*\leftrightarrow NH_2*+H*$, (3) $NH_2*+*\leftrightarrow NH*+H*$, (4) $NH*+*\leftrightarrow N*+H*$, (5) $2N*\leftrightarrow N_2*+*$, (6) $N_2*\leftrightarrow N_2(g)+*$, (7) $2H*\leftrightarrow H_2*+*$, (8) $H_2*\leftrightarrow H_2(g)+*$. It is observed that across all the examined temperature points, perturbing the rate constant of H_2 dehydrogenation step leads to decrease in free active site whereas opposite is observed for NH dehydrogenation step.

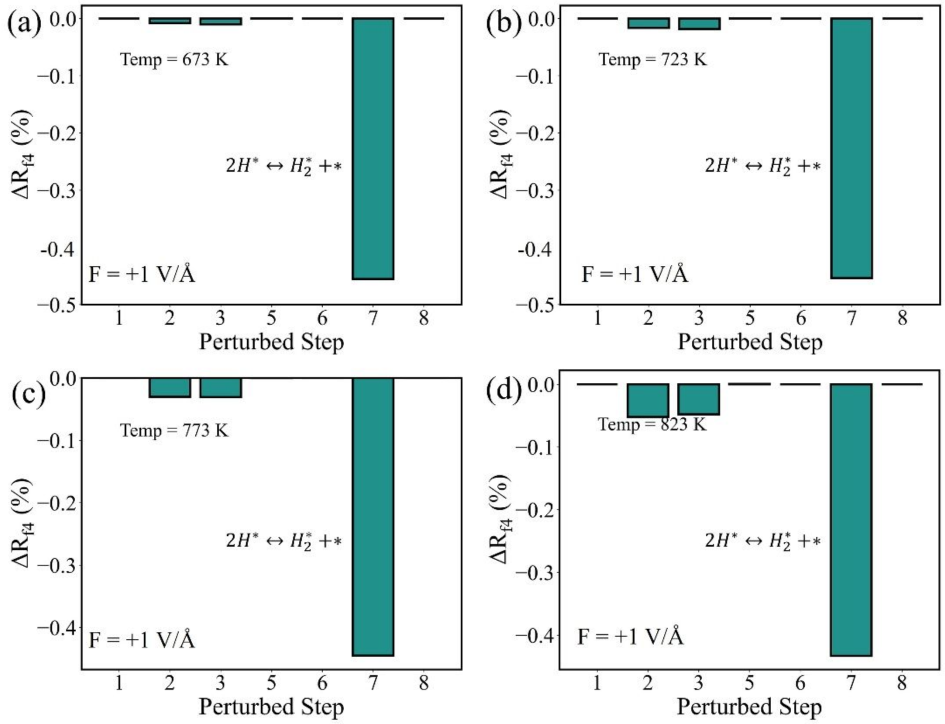

Figure S44. At the applied electric field of +1 V/Å, The impact of a 1% perturbation in the rate constant of each elementary step on the percentage change in the forward rate of the NH dehydrogenation step (Rf4) across the examined temperatures: (a) 673 K, (b) 723 K, (c) 773 K, and (d) 823 K. The corresponding elementary steps numbered in the graph are: (1) NH_3(g)+*↔NH_3*, (2) NH_3*+*↔NH_2*+H*, (3) NH_2*+*↔NH*+H, (5) 2N*↔N_2*+*, (6) N_2*↔N_2(g)+*, (7) 2H*↔H_2*+*, (8) H_2*↔H_2(g)+*. The graph skips plotting the (4) NH*+*↔N*+H* step bar data, as it is intuitive that increasing the rate constant of a step will obviously result into enhancement the rate of that reaction. It is observed that across all the examined temperature points, perturbing the rate constant of H_2 dehydrogenation step leads to reduction in rate of rate-limiting step (R_{f4}).

REFERENCES

1. García-García, F. R.; Guerrero-Ruiz, A.; Rodríguez-Ramos, I., Role of B5-Type Sites in Ru Catalysts used for the NH_3 Decomposition Reaction. *Topics in Catalysis* **2009,** *52* (6), 758-764.
2. Masumian, E.; Hashemianzadeh, S. M.; Nowroozi, A., Hydrogen adsorption on SiC nanotube under transverse electric field. *Physics Letters A* **2014,** *378* (34), 2549-2552.
3. Che, F.; Hensley, A. J.; Ha, S.; McEwen, J.-S., Decomposition of methyl species on a Ni (211) surface: investigations of the electric field influence. *Catalysis Science & Technology* **2014,** *4* (11), 4020-4035.
4. Che, F.; Gray, J. T.; Ha, S.; McEwen, J.-S., Catalytic water dehydrogenation and formation on nickel: Dual path mechanism in high electric fields. *Journal of Catalysis* **2015,** *332*, 187-200.
5. Che, F.; Ha, S.; McEwen, J.-S., Elucidating the field influence on the energetics of the methane steam reforming reaction: A density functional theory study. *Applied Catalysis B: Environmental* **2016,** *195*, 77-89.
6. Che, F.; Gray, J. T.; Ha, S.; McEwen, J.-S., Reducing Reaction Temperature, Steam Requirements, and Coke Formation During Methane Steam Reforming Using Electric Fields: A Microkinetic Modeling and Experimental Study. *ACS Catalysis* **2017,** *7* (10), 6957-6968.
7. Che, F.; Gray, J. T.; Ha, S.; McEwen, J.-S., Improving Ni Catalysts Using Electric Fields: A DFT and Experimental Study of the Methane Steam Reforming Reaction. *ACS Catalysis* **2017,** *7* (1), 551-562.
8. Che, F.; Ha, S.; McEwen, J.-S., Hydrogen Oxidation and Water Dissociation over an Oxygen-Enriched Ni/YSZ Electrode in the Presence of an Electric Field: A First-Principles-Based Microkinetic Model. *Industrial & Engineering Chemistry Research* **2017,** *56* (5), 1201-1213.
9. Che, F.; Ha, S.; McEwen, J. S., Catalytic reaction rates controlled by metal oxidation state: C− h bond cleavage in methane over nickel-based catalysts. *Angewandte Chemie* **2017,** *129* (13), 3611-3615.
10. Fang, H.; Wu, S.; Ayvali, T.; Zheng, J.; Fellowes, J.; Ho, P.-L.; Leung, K. C.; Large, A.; Held, G.; Kato, R.; Suenaga, K.; Reyes, Y. I. A.; Thang, H. V.; Chen, H.-Y. T.; Tsang, S. C. E., Dispersed surface Ru ensembles on MgO(111) for catalytic ammonia decomposition. *Nature Communications* **2023,** *14* (1), 647.
11. Lucentini, I.; Garcia, X.; Vendrell, X.; Llorca, J., Review of the Decomposition of Ammonia to Generate Hydrogen. *Industrial & Engineering Chemistry Research* **2021,** *60* (51), 18560-18611.
12. Stegelmann, C.; Andreasen, A.; Campbell, C. T., Degree of rate control: how much the energies of intermediates and transition states control rates. *J Am Chem Soc* **2009,** *131* (23), 8077-82.
13. Campbell, C. T., The Degree of Rate Control: A Powerful Tool for Catalysis Research. *ACS Catalysis* **2017,** *7* (4), 2770-2779.
